# A novel dominant selection system for plant transgenics based on phosphite metabolism catalyzed by bacterial alkaline phosphatase

**DOI:** 10.1371/journal.pone.0259600

**Published:** 2021-11-04

**Authors:** Hang Yuan, Yuxian Wang, Yanjuan Liu, Mengru Zhang, Zhurong Zou

**Affiliations:** 1 Engineering Research Center of Sustainable Development and Utilization of Biomass Energy, Ministry of Education, School of Life Sciences, Yunnan Normal University, Kunming, Yunnan, China; 2 NHC Key Laboratory of Drug Addiction Medicine, The First Affiliated Hospital, Kunming Medical University, Kunming, Yunnan, China; National University of Kaohsiung, TAIWAN

## Abstract

Selective markers are generally indispensable in plant genetic transformation, of which the frequently used are of antibiotic or herbicide resistance. However, the increasing concerns on transgenic biosafety have encouraged many new and safe selective markers emerging, with an eminent representative as phosphite (Phi) in combination to its dehydrogenase (PTDH, e.g. PtxD). As bacterial alkaline phosphatase (BAP) can resemble PtxD to oxidatively convert toxic Phi into metabolizable phosphate (Pi), herein we harnessed it as the substitute of PtxD to develop an alternative Phi-based selection system. We first validated the *Escherichia coli* BAP (EcBAP) did own an extra enzymatic activity of oxidizing Phi to Pi. We further revealed EcBAP could be used as a dominant selective marker for Agrobacterium-mediated tobacco transformation. Although the involved Phi selection for transformed tobacco cells surprisingly required the presence of Pi, it showed a considerable transformation efficiency and dramatically accelerated transformation procedure, as compared to the routine kanamycin selection and the well-known PtxD/Phi system. Moreover, the *EcBAP* transgenic tobaccos could metabolize toxic Phi as a phosphorus (P) fertilizer thus underlying Phi-resistance, and competitively possess a dominant growth over wild-type tobacco and weeds under Phi stress. Therefore, this novel BAP/Phi-coupled system, integrating multiple advantages covering biosafe dominant selective marker, plant P utilization and weed management, can provide a PTDH-bypass technological choice to engineer transgenic plant species, especially those of great importance for sustainable agriculture.

## Introduction

Selective markers are generally required for plant transgenic studies to enable the putative transformants regenerated under a selection pressure, and can be overall grouped into two categories, the negative selection marker and the positive/dominant selection marker [1,2]. The former type is the most widely used and usually featured with antibiotic/or herbicide resistance [3]. With the increasing concerns on their inherent defects, i.e. the potential threats to ecological environment and food safety [4–6], development of a biosafety-secured dominant selection marker is of great significance and necessity, and always ongoing [7–11].

Phosphite (Phi) is the second phosphorus (P) reserve in Earth, but confers a higher solubility, a lower chemical reactivity with soil components, and a better thermo/photo- stability, as compared to phosphate (Pi) [12]. Moreover, Phi can structurally mimic Pi to enter plant Pi transport system for efficient absorption [13], thus integrally accounting for its higher accessibility to plants. Nowadays, Phi has already been used as a fungicide, pesticide, and biostimulant for plants, with negligible harm to human health and environment [12,14,15]. However, Phi can not be directly applied as a P fertilizer for plants, but rather like a herbicide to inhibit plant growth and development due to its interference with plant Pi signaling pathway [16,17]. In addition, Phi cannot be metabolized by algae [18–21], thus taking no risk of forming algal blooms to threat aquatic ecosystems [22]. Phi, as a reduced form of P source, is assimilated by organisms only if oxidized to Pi [23].

The above properties and advantages of Phi had evoked the establishment of a PtxD/Phi-coupled selection system for plant transgenics [11,24]. In this system, PtxD is a phosphite dehydrogenase (PTDH) from the Phi-autotrophic bacteria *Pseudomonas stutzeri*, which can efficiently catalyze the oxidative conversion of Phi to Pi and the reduction of cofactor NAD^+^ to NADH [25]. PtxD can detoxify Phi (as the selection agent / P pre-fertilizer / herbicide) and turn bad into good, simultaneously accomplishing triple roles of a safe dominant selective marker, plant P utilization and weed management [24,26–28]. This would prevent the potential risks (e.g. uncontrolled biosafety, escaping selection) from the use of traditional antibiotics/herbicides [5,29]. During transformation culturing under Phi selection, the *PtxD*-transformed cells can convert Phi into metabolizable Pi, and thus survive, thrive and dominantly regenerate into green shoots, as compared to the non-transformed ones. When using Phi as a P fertilizer/herbicide, the *PtxD* transgenic plants can metabolize toxic Phi into nutritional Pi, while the wild weeds are retarded by Pi deprivation and die. This might reduce the competitive use of the soil Pi by weeds, largely save the current Pi consumption, and also prevent Pi overuse-incited water eutrophication and algal blooms [12,18–22]. Moreover, the weeds can hardly evolve the Phi-tolerance naturally to escape inhibition [24].

This pleiotropic PtxD/Phi selection system has been shortly exploited to develop transgenic variants for both monocot and dicot plant species including Arabidopsis [24], tobacco [24,30], and other more important crops such as cotton [27], maize [31] and sorghum [32], since its invention several years ago, with fascinating application prospects in sustainable agriculture [11]. Moreover, it has also been extensively used to genetically engineer important microorganisms (e.g. yeast [33], Trichoderma fungi [34]) and microalgae (e.g. Chlamydomonas [35–38], Cyanophyta alga [20,21]) as cost-effective bio-production platforms under non-axenic conditions, owing to Phi-directed control of biological contaminants. Nonetheless, in order to expand the Phi-based biotechnological applications beyond PTDH, a functional alternative to PtxD is certainly desirable.

In this context, we noticed the report of Yang and Metcalf (2004) that the *phoA-*encoded bacterial alkaline phosphatase (BAP) in *E*. *coli* (termed EcBAP) had an additional ability beyond its main phosphate hydrolysis activity, i.e. oxidizing Phi irreversibly to Pi with hydrogen emission [39]. The dual roles of this enzyme depend on the ambient pH, in which the activities of Phi oxidization and phosphate hydrolysis are respectively enhanced by acidity and alkality, and vice versa. BAP is a secretory metal enzyme in periplasm and functions in an oxidized form of homologous dimer containing four essential disulfide bonds [40,41]. BAP has been widely used as an important tool enzyme in molecular cloning and immunoassays, due to its phosphate hydrolysis activity. Surprisingly, the Phi-oxidizing feature of EcBAP seems neglected since its revelation. There was only one related periodical report on EcBAP for Phi use efficiency and weed control in hygromycin-selected transgenic rice plant, but irrelevant to selective marker [42], rightly as done previously for PtxD [43]. Nearly at the same time, we carried out a more comprehensive study on plant transgenics of *EcBAP*, which was now presented herein. First, we focused to test whether or not EcBAP could replace PtxD as a Phi-metabolized dominant selective marker for plant genetic transformation, and then evaluated the Phi-resistance of *EcBAP* transgenic plants under Phi stress. We expected to establish a trinary (selective marker / plant P utilization / weed control) system based on EcBAP/Phi combination, resembling the well-known PtxD/Phi suite, so as to provide an alternative technical choice for plant genetic engineering.

## Results

### Gene cloning of *EcBAP* and construction of its expression vectors

BAP is a secretory enzyme in bacterial periplasm. The N-terminal signal peptide (SP) is not required for its activity. Herein, using *E*. *coli* DH5α genome as the template, we deliberately amplified the coding DNA of EcBAP lacking its SP (1–21 amino acids (aa)). For efficient expression in plants, a Kozak sequence was introduced in the forward PCR primer EcBAP-5Bm, thus imparting *EcBAP* a high translation potential. This amplicon was directly subcloned by dual enzyme (*BamH* I/*Sac* I) digestions in a binary plasmid pBI121 to generate the plant expression vector pBI(EcBAP) (S1 Fig) for subsequent tobacco transformation. The cloned *EcBAP* gene was verified by sequencing, with 100% accuracy in its deduced protein (S2 Fig).

To preliminarily verify the Phi-oxidizing activity of EcBAP, we further constructed its prokaryotic expression vector pET(EcBAP) by transplanting the *EcBAP* gene from pBI(EcBAP) onto the backbone of pET32a(+) via designated enzyme (*Nde* I/*Xho* I) digestions. This recombinant EcBAP (~ 47.2 kDa) has a fused C-terminal His6-tag that is suitable for affinity chromatography purification.

### Expression and purification of the recombinant EcBAP

**Fig 1:**
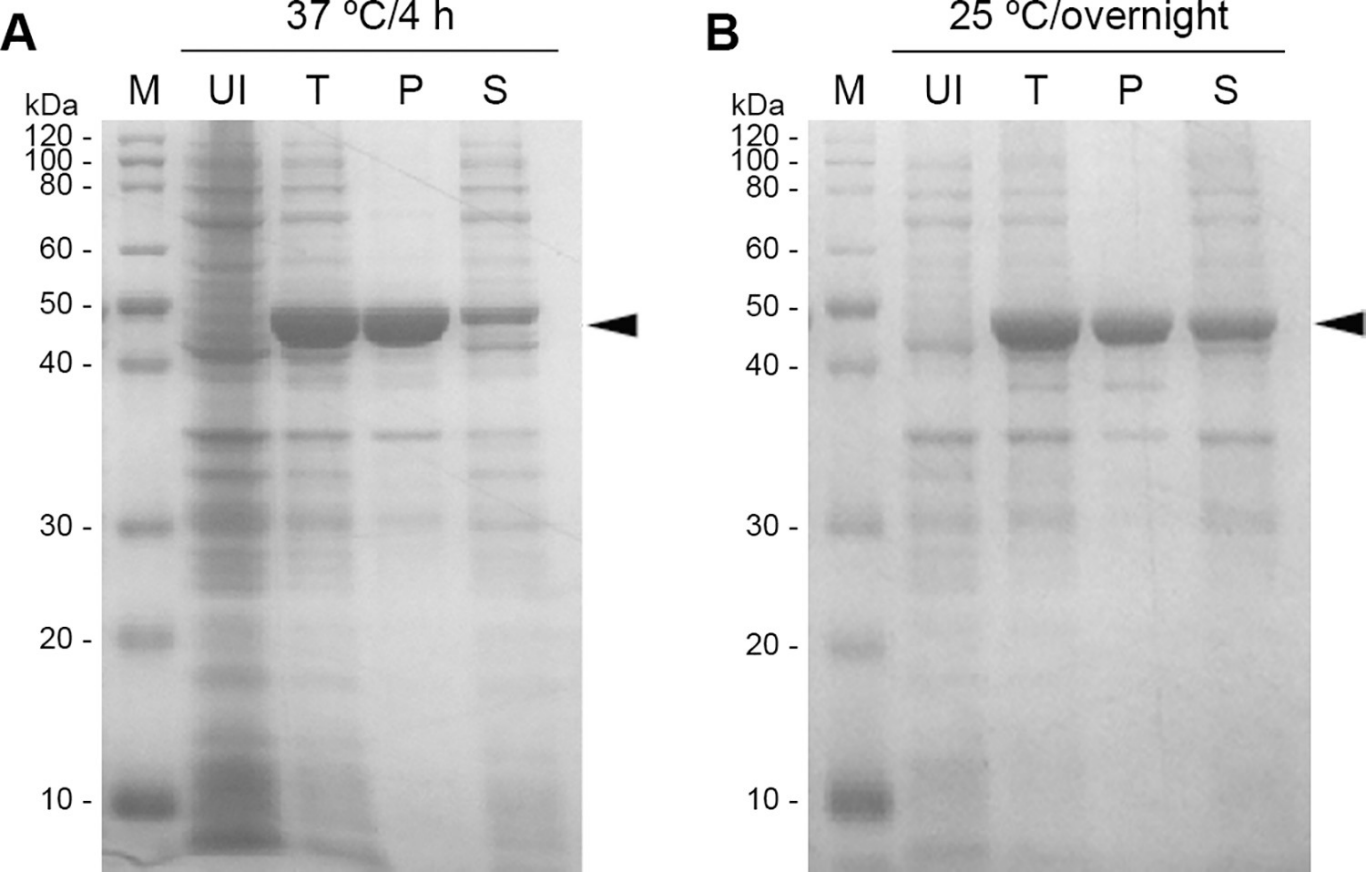
The recombinant expression of EcBAP in *E*. *coli* BL21(DE3) strain. (A) induced at 37°C for 4 h; (B) induced at 25°C overnight. M: Protein marker; UI: the total lysate of uninduced bacterial cells; T: the total lysate of induced bacterial cells; P: the pellet of ‘T’; S: the supernatant of ‘T’; Arrow-head indicates the recombinant EcBAP protein. The original gel images of this figure (A, B) are available in S1 File.

The inducible expression of prokaryotic vector pET(EcBAP) was conducted in a routine *E*. *coli* strain BL21(DE3). Due to the omission of SP, the recombinant EcBAP protein was restrictively located in the cytoplasm. The cell lysates obtained by ultrasonic disruption were analyzed by SDS-PAGE. As seen from Fig 1A and 1B, no matter what the induction at 37°C or 25°C, the recombinant EcBAP demonstrated a robust and correct expression in *E*. *coli*. However, most of the EcBAP protein expressed at 37°C was accumulated in the inclusion bodies with a poor solubility (less than 30%), while its solubility increased significantly (up to 50%) when expressed at 25°C. This is in consistence with the classical viewpoint that the low temperature induction can improve the solubility of target proteins recombinantly expressed in *E*. *coli* [44].

The recombinant EcBAP protein expressed at 25°C could be efficiently purified by His-tag specific immobilized metal affinity chromatography (IMAC) under Pi-free conditions, and almost eluted at one time by 200 mM imidazole solution.

### The recombinant EcBAP has an activity to oxidize Phi *in vitro*

**Fig 2:**
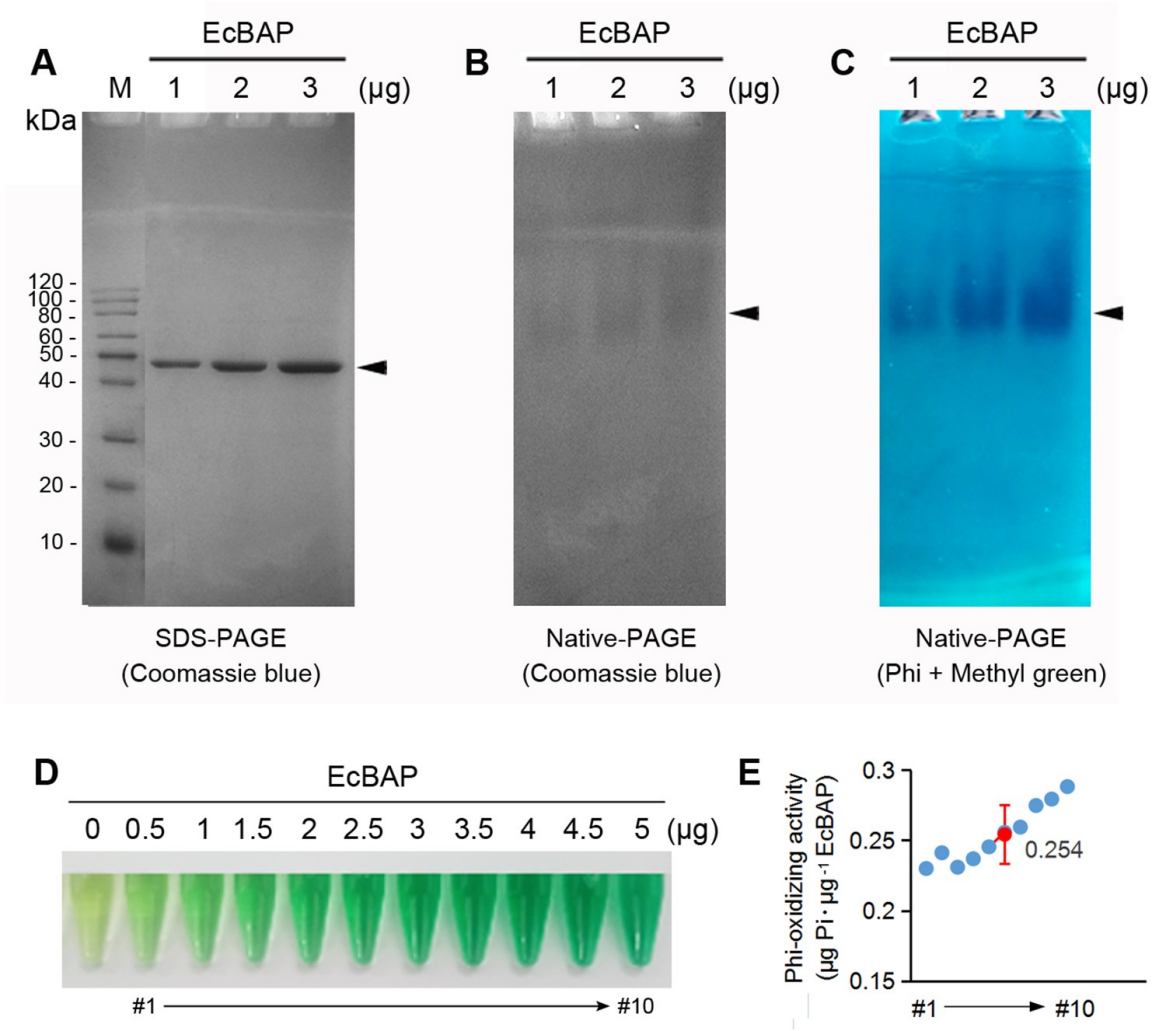
The recombinant EcBAP has an apparent Phi-oxidizing activity *in vitro*. (A) The recombinant EcBAP was analyzed by SDS-PAGE and coomassie blue staining for purity assessment; (B) The recombinant EcBAP at the non-denatured status was visualized by native-PAGE and coomassie blue staining; (C) The Phi-oxidizing activity of recombinant EcBAP was qualitatively evaluated by native-PAGE gel activity staining in a consecutive reactant system composed of Phi and methyl green; (D) Ten individual reactions (#1–#10) were conducted for Pi/AM/MG-based spectrometric assay to quantitatively determine the Phi-oxidizing activity of recombinant EcBAP, and (E) The calculated Phi-oxidizing activity (μg Pi · μg^-1^ EcBAP) of all ten reactions were shown together, with the mean (±SD) marked in red (also see S2 File). Arrow-head indicates the recombinant EcBAP protein. The original gel images for this figure (A–C) are available in S1 File.

The purified EcBAP protein was checked by SDS-PAGE and coomassie blue staining (Fig 2A), with a high homogeneity. Then, a native-PAGE gel with the same sample loading was either used for visualizing the non-denatured EcBAP protein by coomassie blue staining (Fig 2B) or subjected to the activity staining of EcBAP in a system containing the substrate of Phi and the dye of methyl green (Fig 2C). On the native gel, the migrated EcBAP could *in-situ* react with Phi to produce Pi that was immediately fixed by calcium ion. The newly formed insoluble calcium phosphate was then incubated with ammonium molybdate and consequently converted to insoluble phospho-molybdate complex which could be distinctly stained by methyl green to develop blue bands under weak acidic condition (Fig 2C). The migration and band profiles of EcBAP on native gels stained by coomassie blue or methyl green were quite similar, wherein the band overall appeared as a single form and its density was proportional to the sample loading (Fig 2B and 2C). Clearly, this result implies the recombinant EcBAP is able to oxidize Phi to Pi.

We further used a Pi/ammonium molybdate (AM)/malachite green (MG)-based spectrometric assay to estimate the Phi-oxidizing activity of recombinant EcBAP. Ten reactions were applied by incubating 0.5–5 μg (0.5 μg interval) of purified EcBAP with Phi, the newly generated Pi was reacted with AM/MG/T (Tween-20) mixture to form a green-blue conjugate that has a maximum absorption at the wavelength of 660 nm (OD_660_) for measuring Pi amount. As observed from Fig 2D, the developed color was intensified with the increasing usage of purified EcBAP. Finally, the Phi-oxidizing activity of recombinant EcBAP for each reaction was determined, ranging in 0.23–0.288 (0.254 on average) μg Pi · μg^-1^ EcBAP (Fig 2E and S2 File), which is seemingly much lower than that of the native BAP extracted from *E*. *coli* periplasm [39].

### Leaf explant regeneration of kanamycin-selected *EcBAP* transgenic tobacco under Phi stress requires the presence of Pi

Considering the aforementioned *in vitro* Phi-oxidizing activity, EcBAP should be rationally eligible as a Phi-coupled selective marker for plant transformation, generally resembling the potent PTDH-type PtxD. However, the relative low ‘Phi to Pi’ converting activity would undoubtedly incite an inquiry on EcBAP whether or not it could be competent for this role in case of Phi as the sole P source. To clarify this puzzle and understand some prerequisites specific to EcBAP, we first generated the kanamycin (Kan)-selected *EcBAP* transgenic (termed EcBAP(Kan)) tobaccos using NPTII selective marker originally from the binary vector pBI121. The main selection process (T3+T4 stages) averagely lasted 50 days for Agrobacterium-mediated tobacco transformation of pBI(EcBAP) under 100 mg·L^-1^ Kan selection on standard MS medium (S3A Fig). The regenerated EcBAP(Kan) transgenic tobacco plantlets were identified by multiplexed PCR (S3B Fig), with a positive transformation efficiency up to 66.7%.

**Fig 3:**
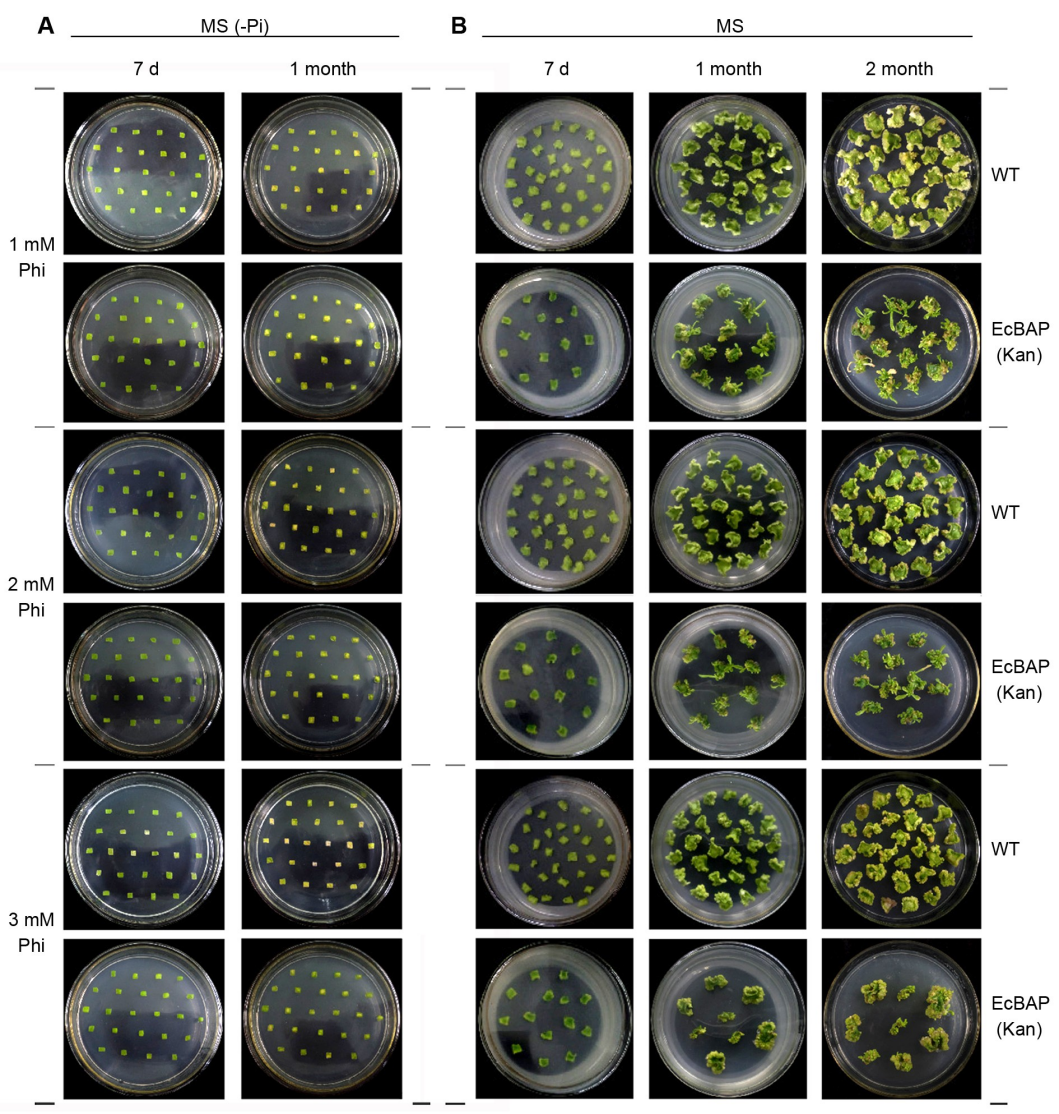
Leaf explant regeneration test of EcBAP(Kan) transgenic tobacco under low to moderate Phi stress. Small leaf pieces (0.5 cm × 0.5 cm) of WT and EcBAP(Kan) transgenic tobacco were pairwise laid on (A) MS (-Pi) or (B) standard MS medium, containing Phi of low to moderate concentrations (1, 2, 3 mM). After 7 days, 1 month, and even 2 months, the differentiation/regeneration status of these leaf explants under Phi stress were photo-recorded and compared between WT and EcBAP(Kan) transgenic tobacco.

Then, the small leaf pieces from these positive transgenic plantlets were directly subjected to a test of leaf explant regeneration on Pi-free MS (i.e. MS (-Pi)) medium containing Phi of different concentrations, using wild-type (WT) tobacco as the control (Figs 3A and S4A). The status of these leaf explants under Phi stress was observed at different time points. After 7 days, WT tobacco leaves were whitened more seriously with the increase of Phi concentration and almost completely at 10 mM Phi, while no obvious chlorosis happened on EcBAP(Kan) transgenic tobacco. Commonly, both were not found with callus differentiation. One month later, WT tobacco leaves became yellow or white without any callus differentiation at each Phi stress, and almost completely whitened at a Phi concentration over 5 mM. In contrast, the overall status of EcBAP(Kan) tobacco leaves was better with much milder whitening, and the leaves were swollen with a sign of callus formation, but no obvious callus buds emerged. These results suggest that the EcBAP(Kan) transgenic tobacco can detoxify Phi to some extent, but surprisingly can not pursue leaf differentiation and regeneration on a medium completely lacking Pi.

We further implemented this test on standard Pi-containing MS medium (Figs 3B and S4B). On the 7th day, both WT and EcBAP(Kan) tobacco leaves became swollen and ready for callus differentiation at various Phi concentrations, wherein the later was more notable. After 1 month under Phi stress, WT tobacco leaves had callus formed, but the callus was somewhat chlorotic and particularly vitrified at the margin. This phenomenon became more obvious with the increase of Phi concentration. In contrast, at any Phi conditions, the leaf explants of in parallel cultivated EcBAP(Kan) transgenic tobacco overall grew well, from which the differentiated callus pieces stayed bright green and more normal buds occurred. Additionally, green shoots were massively regenerated only from EcBAP(Kan) tobacco leaf explants at the stresses of 1 mM, 2 mM Phi, but hardly emerged at a Phi concentration exceeding 3 mM. We extended our observations till 2 months, and found that the yellowing, browning and whitening were more serious with the increase of Phi stress, which was predominant in tobacco leaf explants of WT versus EcBAP(Kan). Moreover, from the leaf callus of EcBAP(Kan) transgenic tobacco, the regenerated shoots could be seen with an increased number only at 1 mM and 2 mM Phi, a few at 3 mM, but nothing at even higher concentrations of Phi.

Taken these results together, a few of implications can be deduced. Phi is toxic to WT tobacco, which is more serious with the increase of usage. Consequently, the leaves of WT tobacco cannot differentiate into regenerative plants in the presence of Phi even on the normal Pi-containing MS medium. Contrastively, EcBAP(Kan) transgenic tobacco can detoxify Phi likely by genetically introduced EcBAP, thus accounting for its outperformance over WT tobacco to tolerate Phi with a better differentiation/regeneration at any Phi stresses. Nevertheless, probably due to the relatively weak Phi-oxidizing activity of EcBAP, the Phi inhibition at high dosage can not be timely alleviated, and the converted Pi from Phi may also not be enough to satisfy the cellular demands in case of Phi as the sole P source. Plausibly, these would explain why the leaf explant regeneration of EcBAP(Kan) transgenic tobacco occurs only at the conditions of low Phi concentrations (e.g. 1–3 mM) and an accompanying supply of Pi from standard MS medium. In conclusion, replenishing Pi generally benefits the leaf differentiation under Phi stress no matter what WT or transgenic tobacco, and seems indispensable for further shoot regeneration happened only on EcBAP(Kan) transgenic tobacco.

### Generation of Phi-selected *EcBAP* transgenic tobacco requires the presence of Pi

**Fig 4:**
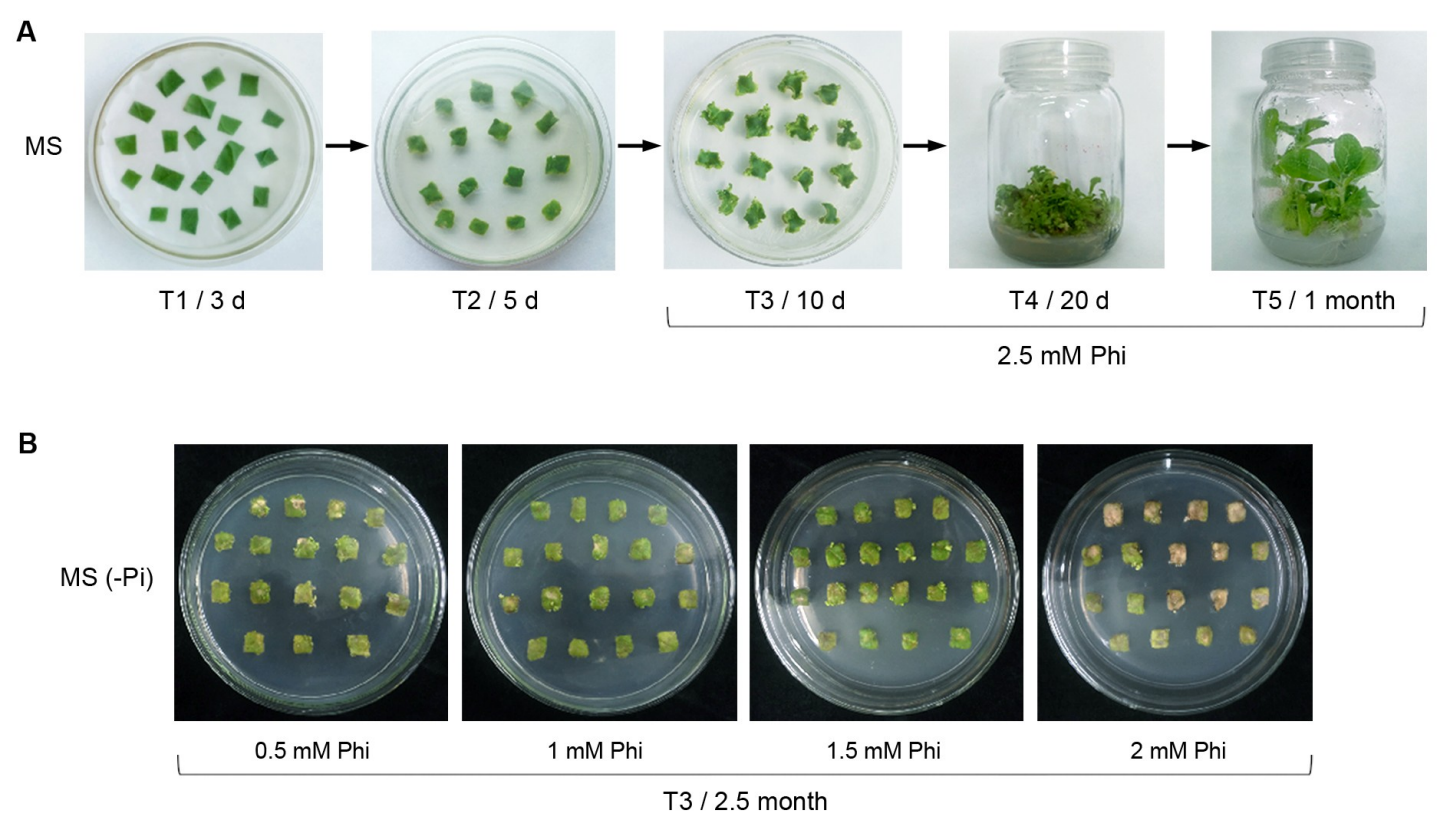
The Agrobacterium-infiltrated tobacco transformation of plant vector pET(EcBAP) under Phi selection. Experiments were performed on (A) standard MS medium and (B) MS (Pi-) medium without Pi supply.

As inspired by above findings, we finally executed Agrobacterium-mediated tobacco transformation of vector pBI(EcBAP) under a suitable selection pressure of 2.5 mM Phi on standard MS medium, using *EcBAP* as the selective marker gene and following a routine procedure (Fig 4A). When comparing to Kan selection (S3A Fig), Phi-resistant shoot transformants could be rapidly regenerated after 10 d cultivation on T3 medium, with half time saved. Additionally, the T4 stage under Phi selection was further shortened to 20 d, thus counting out a total period of 30 d for the main process of *EcBAP* transformation (T3+T4 stages) and saving 20 d on average. This time course is also much shorter (about one half) than that (8–9 weeks) of PtxD/Phi-directed tobacco transformation [24]. Moreover, the Phi-selected *EcBAP* (termed EcBAP(Phi)) transgenic tobacco plants were strictly identified by multiplexed PCR (S5 Fig), with a positive transformation efficiency of nearly 80% which is mildly higher than that (66.7%) of the aforesaid Kan screening and slightly lower than that (> 90%) of PtxD/Phi selection [24]. Meanwhile, we also performed a control experiment, i.e. conducting the transformation selection on Pi-free MS medium containing a serial concentration (0.5, 1, 1.5, 2 mM) of Phi (Fig 4B). As expected, at any Phi selection stresses, no normal shoot transformants were regenerated from the leaf callus explants growing on MS (Pi-) medium at T3 selection stage even as long as 2.5 months, despite a few of whitened buds emerged at the margin. This is well consistent to the findings in aforementioned tests for leaf explant regeneration of EcBAP(Km) transgenic tobacco (Figs 3 and S4). Conclusively, these results imply that EcBAP can be used as a dominant selective marker for plant transformation using Phi as the selection agent, with pronounced advantages at least eminently embodied by a short selection period. However, generation of transgenic plants through the EcBAP/Phi selection system may still need the nutritional nursing of Pi.

### EcBAP(Phi) transgenic tobaccos demonstrate a constitutive *EcBAP* expression

The positive EcBAP(Phi) transgenic tobacco plants were cultivated in greenhouse under normal conditions. Three transgenic lines (termed EcBAP(Phi)- 1, 2, or 6) were chosen for transcriptional expression analysis. As shown in S6A Fig, the total RNA from the main tissues (root, stem, and leaf) of these transgenic plants were qualified for RT-PCR, which was confirmed by the expression profile of an internal reference gene of tobacco, i.e. *18S rRNA* (S6B Fig). Concomitantly, expression of the transgene *EcBAP* at mRNA level was assessed, using WT tobacco as the control. As shown in S6C Fig, the exogenous *EcBAP* could express commonly in the roots, stems, and leaves of all these transgenic lines. Such a constitutive expression of *EcBAP* is in accordance with the general feature of the used *CaMV 35S* promoter.

### EcBAP(Phi) transgenic tobaccos can tolerate Phi toxicity

**Fig 5:**
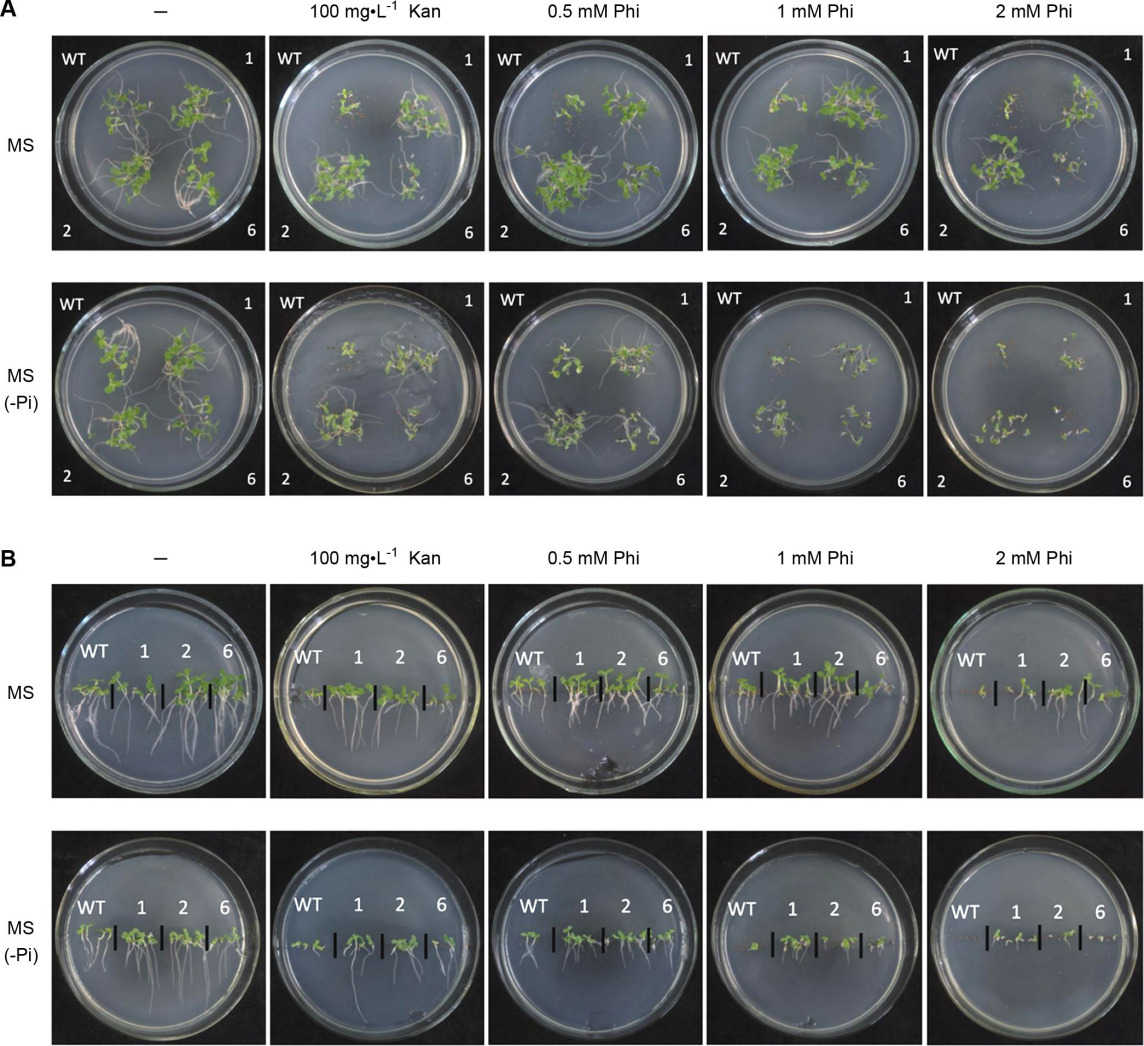
EcBAP(Phi) transgenic tobacco has an evident Phi-resistance, as evaluated by seed germination and seedling growth under Phi stress. The sterilized seeds of WT tobacco and EcBAP(Phi) transgenic lines (1, 2, 6) were sowed on standard MS or MS (-Pi) medium individually containing different concentrations (0.5, 1, 2 mM) of Phi as well as 100 mg·L^-1^ Kan. Their holding petri-dishes were placed (A) horizontally or (B) vertically in a plant growth chamber at normal cultivation conditions for 2 weeks, and then photo-recorded for comparison.

These EcBAP(Phi) transgenic tobacco plants were finally cultivated to flowering and seed-setting, with no aberrant morphology during the full growth. After seed harvest, we performed an evaluation about their Phi-resistance, by judging the seed germination and seedling growth on standard MS or MS (-Pi) medium containing different concentrations of Phi, using WT tobacco as the control (Fig 5). For an intuitive and reliable comparison, we simultaneously implemented two types of plate tests, i.e. placing the seed-sowed petri-dishes horizontally (Fig 5A) and vertically (Fig 5B). On standard MS medium with the stresses of 0.5, 1, 2 mM Phi, the seed germination and seedling growth were remarkably inhibited in WT tobacco with an apparent reduction in germination rate, stem height, root length and leaf green color, which became worse with increased concentration of Phi. Contrastively, this inhibition was less evident in all three selected EcBAP(Phi) transgenic lines (1, 2, 6), despite with an exacerbating tendency under higher Phi stresses. On MS (-Pi) medium, the Phi-induced inhibition at each concentration on seed germination and seedling growth was more serious for either WT tobacco or EcBAP(Phi) transgenic lines (1, 2, 6), as compared to the scenario on standard MS medium. Nevertheless, the EcBAP(Phi) transgenic lines still surpassed WT tobacco, with relatively less deterioration suffered from Phi. Meanwhile, we also compared such inhibition effect of Phi versus 100 mg·L^-1^ Kan, the equivalent of which seemingly sit at a concentration of about 1 mM Phi.

In all, these results demonstrate that the EcBAP(Phi) transgenic tobacco has an obvious tolerance to Phi toxicity, and the presence of Pi can mitigate Phi inhibition to promote seed germination and seedling growth to some extent. Probably, an associated and acceptable explanation might still points to the inherent but mild Phi-oxidizing activity of EcBAP introduced in transgenic tobaccos.

### EcBAP(Phi) transgenic tobaccos demonstrate a dominant growth in a Phi-based weed control experiment

We firstly examined the Phi-tolerance of two surrogate weed species, Bermuda grass and Tall fescue, by sowing the seeds on the filter papers soaked with 0.1 x MS (–P, +80 mg·L^-1^ Pi, or +120 mg·L^-1^ Phi). After 20 days of standard cultivation in a plant growth chamber, the seed germination and seedling growth status were appraised. As seen in S7 Fig, both weed species were remarkably inhibited by Phi, as compared to other two treatments in which Pi supply was additionally more favourable. This is an affirmative that Phi has the potential as a herbicide. Moreover, Tall fescue outperformed Bermuda grass with a better seed vigor, and thus was chosen for further weed control experiments.

**Fig 6:**
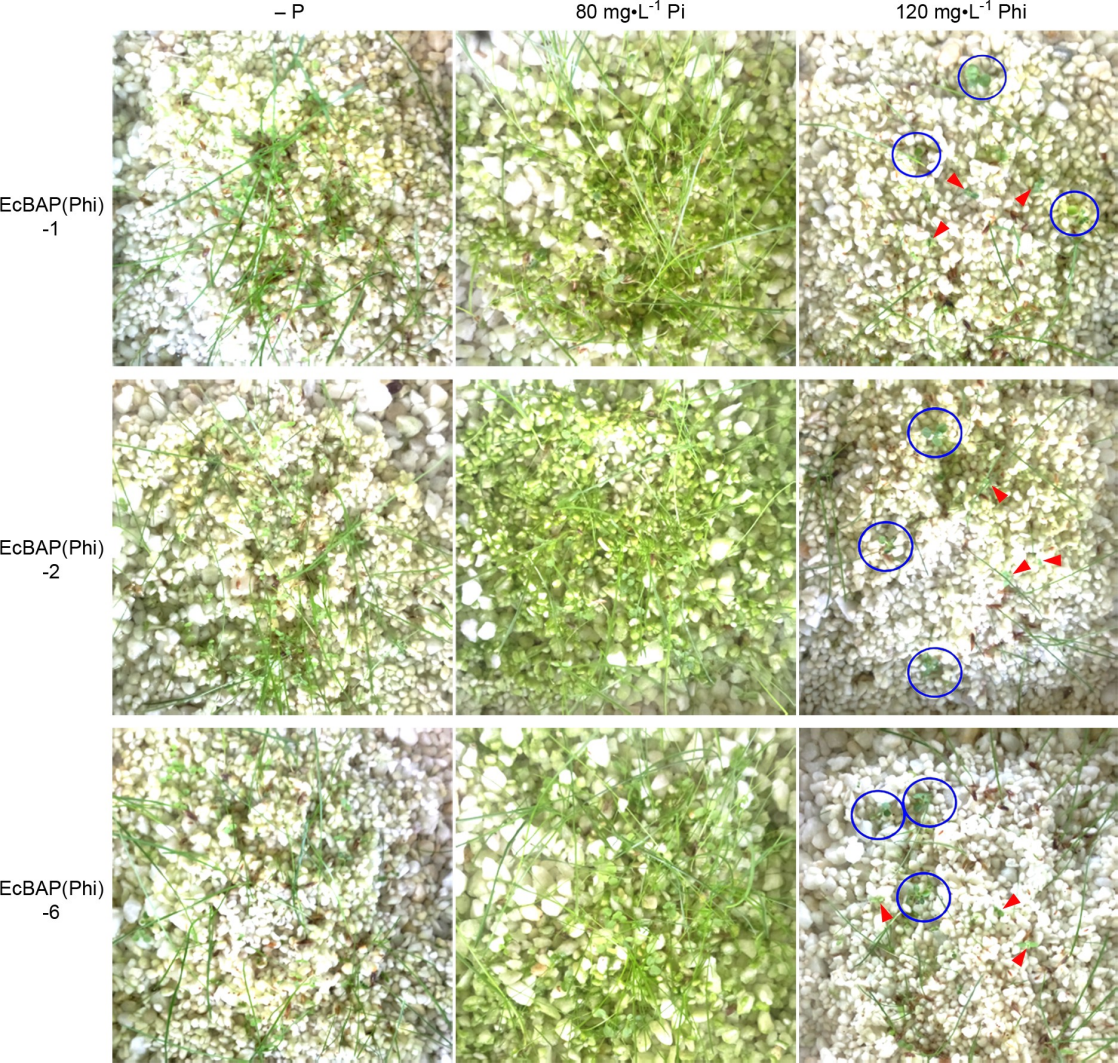
Weed control simulation test by judging the competitive growth of EcBAP(Phi) transgenic tobacco versus weed and WT tobacco. A seed mixture of WT tobacco, EcBAP(Phi) transgenic tobacco line (1, 2, or 6) and Tall fescue weed (at a ratio of 1:1:4) was evenly sowed on a matrix composed of perlite, vermiculite and little gravel, which was individually irrigated with 0.1 x MS (–P, +80 mg·L^-1^ Pi, or +120 mg·L^-1^ Phi). After 15 days of standard cultivation in greenhouse, the growth status of mixed seedlings was photo-recorded and compared. Under the condition of 120 mg·L^-1^ Phi, the growth-dominant tobacco seedlings were circled in blue, while those inhibited individuals were marked by red arrow-heads.

Then, we sowed a seed mixture of Tall fescue weed, WT tobacco and EcBAP(Phi) transgenic tobacco line (1, 2, or 6) on a solid matrix composed of perlite, vermiculite and little gravel, individually watered with 0.1 x MS (–P, +80 mg·L^-1^ Pi, or +120 mg·L^-1^ Phi). After 15 days of cultivation in greenhouse, the growth status of mixed seedlings was reciprocally compared (Fig 6). Therein, that with Pi supply overall ranked as the best, while P-free irrigation as the second and Phi application as the last, reinforcing that Pi has a beneficial but Phi has a strong inhibitory effect on plants no matter what the tobaccos or weeds. The Pi nourishing even induced an astonished contamination of ‘airborne’ microalgae (most likely as Cyanophyta alga), while a sparse density of seedlings and few algal contaminants were only tied to Phi as well as the P absence. However, when re-scrutinizing the Phi-watered tobacco seedlings, some were distinguishedly larger (circled in blue) than those indicated by red arrow-heads (Fig 6). Through a strict PCR identification as aforementioned, these growth-dominant individuals all were substantiated as the expected EcBAP(Phi) transgenic lines (S8 Fig). Therefore, it can be inferred that the EcBAP(Phi) transgenic tobaccos confer a competitive growth with definite Phi-resistance, which is presumably ascribed to the heterogenously introduced EcBAP enzyme that can oxidatively convert the toxic Phi to nutritional Pi. The non-transgenic weeds and WT tobaccos as well as the microalgae are sensitive to Phi inhibition, indicating that Phi is really eligible as a herbicide and algicide. Conclusively, it is feasible to develop a weed control system based on EcBAP/Phi combination, simultaneously with an extra possibility to avoid the risks of algal blooms.

## Discussion

Nowadays, developing transgenic crops is of great importance and increasingly of more attention in modern agriculture [45]. However, the biosafety associated with the residual marker genes in traditional plant genetic transformation remains as an onerous challenge [4–6]. In recent years, an exquisite solution has been hallmarked by the PtxD/Phi system using PtxD as the dominant selective marker and Phi as the selection agent [11,24]. Additionally, *PtxD* transgenic plants can convert toxic Phi into bio-utilizable Pi, while non-transgenic plants and wild weeds do not own the ability to metabolize Phi, thus allowing Phi can play an extra dual role as a new P-fertilizer and herbicide [26]. Therefore, the PtxD/Phi system is indeed advantaged with an aggregate of safe selective marker, plant P utilization and weed management, and has emerged as a powerful Agro-biotechnology with splendid application prospects [11,12,24,26–28]. As BAP has a Phi-oxidizing activity similar to that of PtxD [39], herein we investigated whether it could replace PtxD to establish an analogous BAP/Phi system, particularly keeping an eye on its use as a selective marker.

We first evaluated the *in vitro* Phi-oxidizing activity of *E*. *coli* BAP (EcBAP) with the removal of signal peptide. The recombinant EcBAP could be robustly expressed in *E*. *coli* cytoplasm, despite its prototype as a secretory enzyme in periplasm. The bad solubility of recombinant EcBAP could be remarkably improved by lowering the induction temperature (Fig 1), and turned suitable for protein purification by His-tag specific IMAC. The Phi-oxidizing activity of purified recombinant EcBAP was initially validated by gel activity staining and further determined by a spectrometric assay (Fig 2). However, the obtained average specific activity seems quite lower than that of the purified periplasm EcBAP reported by Yang and Metcalf (2004) [39], probably due to the different percentages of active form. The native EcBAP is actually a homo-dimer formed upon oxidation in periplasm [40,41]. Contrastively, the recombinant EcBAP in the reducing environment within *E*. *coli* cytoplasm is conjectured mainly as the proenzyme monomer, thus hindering its natural toxicity (i.e. phosphate hydrolysis activity) to cells and allowing high accumulation (Fig 1 and ref. 41). After cell lysis, the air exposure might enable a minority of recombinant EcBAP oxidized spontaneously into the active dimer, accounting for its measurable activity (Fig 2D and 2E). Nevertheless, we would rather prefer another speculation that the recombinant EcBAP in reduced monomer has an intrinsic but basal Phi-oxidizing activity (Fig 2A–2C) that is harmless to *E*. *coli* cells, and should be fully reactivated when submitting to an oxidative condition. Moreover, this viewpoint can be supported to some extent by an *in vivo* evidence that the recombinant *E*. *coli* cells overexpressing a cytoplasm-located EcBAP demonstrated an increased Phi-resistance [42].

Probably due to an uncertainty on the competence of EcBAP as a selective marker, we preliminarily generated kanamycin-selected EcBAP(Kan) transgenic tobaccos (S3 Fig), and assessed their Phi-tolerance by leaf explant regeneration test (Figs 3 and S4). Overall, the leaf explants of EcBAP(Kan) transgenic tobacco had a stronger tolerance to Phi than those of WT tobacco no matter on standard or Pi-free MS medium, presumably due to the oxidative detoxification of Phi by genetically introduced EcBAP. In addition, only the transgenic tobacco could regenerate Phi-resistant normal shoots from the leaf explants, which was however allowed selectively under low concentration (below 3 mM) of Phi stress and on standard MS medium. The inferred relatively low Phi-tolerance and Pi requirement for shoot regeneration is likely linked to the mild Phi-oxidizing activity of heterologous EcBAP. Thus, the toxicity of Phi at high dosage can not be timely dismissed, the acquired Pi from Phi oxidation may not satisfy the cellular P demands in case of Phi as the sole P source, and shoot regeneration is eventually in need of Pi supply.

Therefore, we followed this antecedent ‘transformation simulation’ and conducted the routine tobacco transformation directly using EcBAP as the selective marker, in which the selection was kept under a suitable pressure of 2.5 mM Phi on standard MS medium (Fig 4A). The main transformation process (i.e. the total selection period till shoot regeneration) only spent 30 days on average, and the positive transformation efficiency nearly reached 80%. These results explicitly manifest a large feasibility of the new EcBAP/Phi selection system in plant transformation, along with pronounced advantages in terms of time and efficiency obviously surpassing the routine NPTII/Kan selection. In addition, the requirement of Pi presence for EcBAP/Phi selection was substantiated by a control transformation experiment on Pi-free MS medium without any shoot transformants emerged (Fig 4B), reinforcing the findings in those leaf explant regeneration tests (Figs 3 and S4). However, such an accessory Pi nourishment can not be regarded as a technical bug, as it can enable *EcBAP*-transformed cells differentiate/regenerate more vigorously into shoots under Phi selection. Furthermore, we would also like to compare our results with the PtxD/Phi selection system previously used in the same tobacco transformation [24]. Therein, the selection was performed under 1 mM Phi stress on Pi-deprived MS medium, and generally last 8–9 weeks for obtaining Phi-resistant shoots suitable for rooting cultivation. Obviously, the PtxD/Phi selection has a long time course almost doubling that of our experimental system. Nevertheless, its unique independence of Pi supply is plausibly ascribed to the more potent ability of PtxD to professionally detoxify Phi into useful Pi, when comparing to our surveyed EcBAP that never succeeded in regenerating Phi-resistant shoot transformants on Pi-free MS medium even at similar conditions (i.e. a low to moderate stress of 0.5–1 mM Phi, a selection period of 2.5 month) (Fig 4B). Additionally, another difference is particularly noteworthy, i.e. a strict Phi stress (2.5 mM) used in our EcBAP/Phi selection to prevent the escape of pseudo-transformants caused by existent Pi nourishment, while a moderate Phi stress (1 mM) applied in PtxD/Phi system to allow the regeneration of more Phi-resistant shoots that are inherently difficult to develop from Pi-free medium (with Phi as the sole P source) but of high positive percentage [24]. Presumably, this comparison should be instructive for their future applications. The use dosage of Phi, the conterbalance between the toxicity of Phi and its derived Pi acquisition, whether or not and how much to pre-add Pi, as well as the transformation duration and efficiency should be deliberately considered, when choosing either of the selection systems. For instance, if using the PtxD/Phi system, an extra and suitable supply of Pi during transformation selection may be especially favourable for those transformation/regeneration-recalcitrant crop species [31,32].

Subsequently, we checked the Phi-resistance of our Phi-selected *EcBAP* transgenic tobaccos. As compared to WT tobacco, all examined transgenic lines showed an evident Phi-resistance, with a remarkably weaker inhibition on seed germination and seedling growth under any concentrations of Phi no matter on standard or Pi-free MS medium (Fig 5). Meanwhile, under the same Phi stress, the inhibition was significantly alleviated by the added Pi for both WT and transgenic tobaccos, consistent with the roles of Pi supply as aforementioned (Figs 3 and 4). We further extended this resistance evaluation in a weed control simulation test. Therein, when comparing to P-free or Pi watering, all transgenic lines with Phi irrigation could be phenotyped with a dominant seedling growth, outcompeting the co-sowed WT tobacco and Tall fescue weed of dwarfed and sparse individuals (Fig 6). In this case, Phi actually effectuated as a herbicide to deteriorate non-transgenic plants including weeds, but was oxidatively detoxified as the utilizable Pi to fertilize *EcBAP* transgenic lines resultantly conferring Phi-resistance and a competitive growth. Thoughtfully, this role switch of Phi should be triggered by the introduced EcBAP constitutively expressed in transgenic tobaccos (S6 Fig). Generally, these results are in accordance with a recent report about the Phi-based fertilization and weed management for hygromycin-selected *BAP* transgenic rice plants [42]. In addition, much less ‘airborne’ microalgae (most likely as Cyanophyta alga) contaminants were also observed under Phi irrigation when comparing to Pi-watering (Fig 6), which could be recognized as an unexpected alga control test in our experiment. Honestly, it does underscore the natural inability of algae to metabolize Phi and the potential of Phi as an alternate P fertilizer to reduce algal blooms and prevent eutrophication [18–22].

Taken together, we can deduce that EcBAP is suitable as a dominant selective marker to generate Phi-resistant transgenic plants that can convert the herbicidal Phi into nutritional Pi for their own use. The triple roles of Phi as a transformation selection agent, P fertilizer and herbicide are concentrically governed by the Phi-oxidizing activity of heterologous EcBAP in its transgenic plants. This enzymatic activity reported first on the native EcBAP [39], was definitely confirmed on its recombinant form *in vitro* (Fig 2) and *in vivo* [42], despite in a reductive and activity-compromised monomer. In plants, the endogenous phosphatases probably own the main phosphate hydrolysis activity, like other eukaryotic orthologs such as animal phosphatases, e.g. calf intestinal phosphatase (CIP) and shrimp alkaline phosphatase (SAP) [39]. Contrastively, their secondary Phi-oxidizing activity is likely missing, thus providing an explanation for the inability of plants to assimilate Phi naturally. However, some prokaryotic BAPs seem evolved bidirectionally with both enzymatic activities dynamically coexisted, in which the Phi-oxidizing activity is enhanced at acidic pH [39]. In most cases, plant cytoplasm is an acidic and redox-homostatic milieu, so it can be envisioned that the *E*. *coli* BAP (i.e. EcBAP) expressed in transgenic tobacco has the chance to oxidatively form the authentic ‘active dimer’ [40,41] and does effectuate well in the enzymatic activity of Phi oxidative metabolism, and thus endue plants the Phi-resistance.

## Conclusion

In summary, our presented work was accomplished according to a rational scheme, with achievements more than the expectation (S9 Fig). To our knowledge, this seems the first demonstration that EcBAP can substitute the precedent PtxD as a dominant selective marker coupled with Phi selection for plant transgenic studies, with a considerable transformation efficiency and dramatically accelerated transformation procedure. Although an accessory supply of Pi is required for EcBAP to effectuate well by compensating its putatively plain Phi-oxidizing activity, it might be commonly favourable when using the PtxD/Phi selection system. Furthermore, EcBAP can also resemble PtxD to drive the Phi-based P fertilization, weed management and even alga control. Therefore, this novel EcBAP/Phi system can provide an alternate route to create desired transgenic plants, especially those important for sustainable agriculture. Prospectively, it might also be applicable in developing engineered microorganisms and microalgae for cost-effective biosynthesis platforms.

## Materials and methods

### Gene cloning and vector construction of *EcBAP*

For the main purpose of this study, the gene cloning and expression vector construction of *EcBAP*, as the initial but essential step, was finished simply by bacterial genomic PCR and the routine molecular operations through restriction digestion and ligation. In detail, by using primers EcBAP-5Bm, EcBAP-3Sc (Table 1) and Phusion high fidelity DNA polymerase (NEB, USA), the gene fragment of the mature EcBAP (lacking SP (1–21 aa)) was amplified from the *E*. *coli* DH5α genome. Then, the purified PCR product was subcloned into plasmid pBI121 (Clontech, USA) by dual digestions with *BamH* I and *Sac* I (NEB, USA) to create the plant expression vector pBI(EcBAP) that was further verified by sequencing with primers 35sPro-Fw, NosDw-Rv (Table 1) derived from *CaMV 35S* promoter and *nos* terminator, respectively.

**Table 1:** Primers used in this study.

Name	Sequence (5’→3’) ^a^	Restriction enzyme
**EcBAP-5Bm**	GTATCGGATCCAGGAGACGCAACAATGGCTACACCTGAAATGCCTGTTCTGGAA	*BamH* I
**EcBAP-3Sc**	GCGTTGAGCTCGGCAGCGAAAATTCACT	*Sac* I
**35sPro-Fw**	GACGTAAGGGATGACGCACAATC	
**NosDw-Rv**	GGATGTGCTGCAAGGCGATTAAGTTG	
**EcBAP-5Nd**	GGCTACATATGGCTACACCTGAAATGCCTG	*Nde* I
**EcBAP-3Xh**	GGATTCTCGAGTTTCAACCCCAGAGCGGCTTTCAT	*Xho* I
**Nt18S-iFw**	GAAACGGCTACCACATCCAAG	
**Nt18S-iRv**	GGCAAATGCTTTCGCAGTTG	
**EcBAP-iFw**	GCGTGGTTATCAGTTGGTGAG	
**EcBAP-iRv**	GTGACTATGACCAGCGTGTTAC	

^a^ The restriction enzyme sites are underlined, the Kozak sequence is boxed, and the translation start codon is shadowed.

Subsequently, the *EcBAP* gene was re-amplified from plasmid pBI(EcBAP) by primers EcBAP-5Nd, EcBAP-3Xh (Table 1) and Phusion DNA polymerase. This amplicon was then subcloned into plasmid pET32a(+) (Novagen, USA) by *Nde* I and *Xho*I (NEB, USA) digestions to construct the prokaryotic expression vector pET(EcBAP).

### Recombinant expression of EcBAP in *E*. *coli*

*E*. *coli* expression is the major source for obtaining large amount of recombinant proteins and usually uses the Novagen pET System. Herein, *E*. *coli* strain BL21(DE3) (Novagen, USA) harboring vector pET(EcBAP) was grown to an exponential OD_600_ of 0.6 in LB medium (containing 100 mg·L^-1^ ampicillin) at 37°C, then induced by 0.5 mM isopropy-β-D-thiogalactoside (IPTG) (Sigma, USA) at 37°C for 4 h or 25°C overnight. A 14 mL culture was precipitated by centrifugation to collect bacterial cells that were subsequently resuspended in 4 mL of 100 mM PBS buffer (pH 7.4) and ultrasonificated for lysis. Each aliquot (e.g. 16 μL) of the crude cell lysate (T) was then partitioned by centrifugation into the supernatant (S) and pellet (P) that was resuspended in the same initial volume of PBS buffer. Meanwhile, a 150 μL cell culture taken before induction was precipitated and resuspended in 16 μL PBS buffer as the uninduced sample (UI). Finally, all these samples were subjected to 12% SDS-PAGE. The solubility of recombinant proteins was estimated by gel band grey-densitometry via the program “Quantity One” (Bio-Rad, USA).

### Purification and Phi-oxidizing activity analysis of the recombinant EcBAP

As the product of Phi oxidation is Pi, this enzymatic activity in EcBAP can be evaluated by the known Pi-specific gel activity staining and spectrometric assay. To avoid any Pi contamination during the purification and activity-analysis of the recombinant EcBAP, all used reagents were required to be free of Pi and the involved equipments (e.g. electrophoresis apparatus) were washed repeatedly with ddH_2_O.

The recombinant expression of EcBAP at 25°C was conducted as mentioned above. The precipitate of a 14 mL induced culture was thoroughly washed with 5 mL Pi-free lysis buffer (20 mM MOPS, 10% glycerol, 1 mM PMSF, 10 μM Leupeptin, pH 7.25), and then resuspended in 4 mL of the same lysis buffer for ultrasonic disruption. Afterwards, the recombinant EcBAP protein in the supernatant of cell lysate was purified by using the routine Ni-NTA IMAC and the column of HisTrap FF crude (GE healthcare, USA), and finally eluted in 200 mM imidazole solution. The purification quality was evaluated on samples with a gradient of aliquots (10, 20, 30 μL) by 12% SDS-PAGE. The concentration of purified EcBAP was determined by using a BCA Protein Assay Kit (Thermo Scientific, USA).

Afterwards, the gel activity staining analysis of recombinant EcBAP was performed according to a modified protocol of Stochaj and Berkelman (2006) [46]. At first, two identical 12% native-PAGE with the aforesaid sample loading of purified EcBAP were parallelly run in a specific electrophoresis buffer (5 mM HEPES, 43 mM imidazole, pH 7.1). Then, one gel was directly stained in coomassie brilliant blue solution for EcBAP protein visualization, and another gel was subjected to gel activity staining as follows: wash twice with ddH_2_O; incubate in a reactive solution (100 mM Tris, 10 mM Na_2_HPO_3_·5H_2_O, 50 mM CaCl_2_, pH 8.5) for 30 min; transfer into 1% ammonium molybdate solution and gently shake for 10 min, repeat this step once; transfer into the staining solution (0.5% methyl green, 7% acetic acid) and mildly shake for 30 min; finally rinse with ddH_2_O and store therein.

Meanwhile, a quantitative malachite green-based spectrometric assay on the enzymatic activity of recombinant EcBAP was also carried out, as previously described [47] with small modifications. Prior to the experiment, a tripartite mixture (AM/MG/T) was freshly prepared by adding 200 μL Tween-20 into 10 mL AM/MG solution that is composed of AM (4.2% ammonium molybdate, 4 M HCl) and MG (0.045% malachite green) at a ratio of 1:3. Each enzymatic reaction was set by adding 5 μL reactive buffer (50 mM MOPS, 10 mM Na_2_HPO_3_·5H_2_O, pH 7.0), 0–50 μL (5 μL increase gradually) of the purified EcBAP solution and ddH_2_O to a total volume of 500 μL, and incubated in a water bath of 37°C for 30 min. Then, 1 mL of the AM/MG/T mixture was added in each reaction for color development. Half hour later, the absorption at the wavelength of 660 nm (OD_660_) of these reactions were measured in a spectrometer (Thermo Scientific, USA). The value of OD_660_ was used to calculate the Pi yield for each reaction, according to a standard Pi curve. The enzymatic activity of recombinant EcBAP was determined by Pi production versus the protein amount of EcBAP, and finally averaged.

### Agrobacterium-infiltrated tobacco transformation

The Agrobacterium-mediated leaf disc transformation of tobacco (*Nicotiana tabacum* L. *cv Xanthi*) [48] was prevalently used for plant transgenic studies (certainly including the evaluation of new selective markers [24]), and thus also conducted herein. Briefly, the small pieces (~1 cm^2^) of aseptic WT tobacco leaves were incubated for 5 min in the culture of *A*. *tumefaciens* strain LBA4404 harboring the pBI(EcBAP) expression vector, then washed three times with sterile ddH_2_O, and placed on T1 co-cultivation medium under 28°C and darkness for 3 d. Afterwards, leaf explants were transferred to T2 bacteria-removing medium (containing 500 mg·L^-1^ carbenicillin (Car)) for 5 d, then to T3 selection medium supplemented with 100 mg·L^-1^ Kan or 2.5 mM Phi. After a period, the resistant calli with emerged small shoots were translocated to T4 subculture medium for regeneration of more shoots. Finally, the green shoots of qualified size were excised from the explants and cultivated in T5 rooting medium for growing into plantlets. From T2 to T5 stage, the culturing in a plant growth chamber was maintained at a normal condition of 26°C, 75% humidity, 1500 lux light intensity, and a regime of 16 h light / 8 h dark. The T1–T5 mediums were prepared using standard Murashige-Skoog (MS) medium (Sigma, USA) and detailed in Table 2. To prepare their Pi-devoid (i.e. MS (-Pi)) forms, a modified MS formula without N, P and K elements was used and re-supplemented with the original content of N and K. The Pi and Phi solutions were prepared using chemicals Na_2_HPO_4_·12H_2_O and Na_2_HPO_3_·5H_2_O, respectively.

**Table 2:** MS-based culture medium used in different stages of Agrobacterium-mediated tobacco transformation under selection of Kan or Phi.

Medium	6-BA (mg·L^-1^)	NAA (mg·L^-1^)	Car (mg·L^-1^)	Kan (mg·L^-1^) / Phi (mM)
**T1 co-cultivation medium**	1	0.1	–	–
**T2 bacteria-removing medium**	1	0.1	500	–
**T3 selection medium**	1	0.1	–	100 / 2.5
**T4 subculture medium**	1	0.1	300	100 / 2.5
**T5 rooting medium**	–	–	100	100 / 2.5

## PCR identification of *EcBAP* transgenic tobaccos

Due to the simplicity, rapidness, accuracy and low cost, PCR has become a routine method to identify transgenic organisms. Herein, total genomic DNA was extracted from the leaf samples of the regenerated tobacco transformants growing in T5 medium-contained glass bottles at the growth chamber or in soil-filled pots at the greenhouse, and then used as the template for multiplexed PCR identification (to strictly avoid the pseudo-positive) of Kan/or Phi- selected *EcBAP* transgenic tobacco plants, with reciprocally combinatorial primer-pairs derived from the coding region of *EcBAP* and the backbone of vector pBI121, e.g. EcBAP-5Bm/EcBAP-3Sc, 35sPro-Fw/EcBAP-3Sc, and EcBAP-5Bm/NosDw-Rv (Table 1).

### Leaf explant regeneration of Kan-selected *EcBAP* transgenic tobacco under Phi stress

To preliminarily evaluate a candidate selective marker, leaf explant regeneration test under the corresponding selection stress can be used as a ‘transformation simulation’ to assess its resistance [24]. This tactic was particularly needed for EcBAP and implemented as below. Aseptic small leaves were excised from WT and the initial (T0) Kan-selected *EcBAP* transgenic (i.e. EcBAP(Kan)) tobacco plantlets growing in glass bottles, then cut into small pieces (0.5 cm × 0.5 cm), and pairwise laid on MS or MS (-Pi) medium containing Phi of different concentrations (1, 2, 3, 4, 5, 10 mM). After 7 days, 1 month, and even 2 months of cultivation in a plant growth chamber with normal condition settings, the differentiation/regeneration status of these leaf explants under Phi stress on standard MS or MS (-Pi) medium were photographically recorded and compared between WT and EcBAP(Kan) transgenic tobacco.

### *EcBAP* expression in transgenic tobaccos analyzed by reverse transcription PCR (RT-PCR)

Transgene expression is the prerequisite for its putative ‘gain of function’, which should be at least checked at the point of transcription. Herein, *EcBAP* expression in its transgenic tobaccos was only qualitatively analyzed by the classic RT-PCR. First, total RNA from the main tissues (root, stem, and leaf) of three Phi-selected *EcBAP* transgenic tobacco lines (EcBAP(Phi)-1, 2, 6) were extracted using the Trizol reagent (Invitrogen, USA). By using M-MLV RTase (Promega, USA) and random primers, aliquots of the RNA samples were then reversely transcribed as the cDNA templates for PCR examination of *EcBAP* transcriptional expression in the main tissues of tobacco by primer-pair EcBAP-iFw/EcBAP-iRv (Table 1). Meanwhile, the tobacco *18S rRNA* gene, as the internal gene expression reference, was analyzed with its specific PCR primer-pair Nt18S-iFw/Nt18S-iRv (Table 1).

### Phi-resistance analysis of *EcBAP* transgenic tobaccos

To examine the acquired traits (i.e. Phi-resistance) imparted by the transgene *EcBAP* in tobaccos, an intuitive and reliable method was thought as the evaluation of seed germination and seedling growth under Phi stresses. Briefly, the sterilized seeds of WT tobacco and transgenic lines (EcBAP(Phi)-1, 2, 6) were sowed individually at four delineated quarters of each petri-dish containing standard or Pi(-) MS medium supplemented with 100 mg·L^-1^ Kan or a serial concentration (0.5, 1, 2 mM) of Phi. These petri-dishes were horizontally placed for seed germination in a plant growth chamber under normal conditions. Meanwhile, a similar test was performed with only small variations, i.e. evenly dot-sowing the different seeds with marked boundaries in one line on each petri-dish and placed them vertically. After 2 weeks, the seed germination and seedlings growth status were photo-recorded and compared between *EcBAP* transgenic lines and WT tobacco.

### Phi-tolerance test of weeds

For a Phi-based weed control test, the basal Phi-tolerance of two candidate wild weed species (Bermuda grass and Tall fescue) was preliminarily assessed. Their seeds were pairwise sowed on each filter paper individually soaked with 0.1 x MS (–P, +80 mg·L^-1^ Pi, or +120 mg·L^-1^ Phi). The holding glass plates (15 cm x 15 cm) were placed in a plant growth chamber under normal conditions. After 20 days of cultivation, the seed germination and seedling growth on each plate were photo-recorded for both horizontal and vertical views.

### Weed control simulation test

To probe the herbicidal role of Phi for future field cultivation of *EcBAP* transgenic tobaccos, a simulation test for weed control was done as briefed below. Uniform seeds of Tall fescue weed, WT tobacco and anyone EcBAP(Phi) transgenic tobacco line (1, 2, or 6) were mixed at a ratio of 4:1:1, and evenly sown in plastic plates containing solid matrix composed of perlite, vermiculite and little gravel. Each suite of sowing was individually irrigated with 0.1 x MS (–P, +80 mg·L^-1^ Pi, or +120 mg·L^-1^ Phi). After 15 days of cultivation in greenhouse, the growth of mixed seedlings on each plate was photo-recorded and compared.

### Statistics

The solubility of recombinant EcBAP was estimated by band grey-densitometry on SDS-PAGE gels from three separate expression experiments. The Phi-oxidizing activity of recombinant EcBAP was measured as the mean±SD from malachite green-based spectrometric assays on triplicate groups of enzymatic reactions. Positive transformation efficiency using Kan or Phi selection was determined as the percentage of PCR-identified *EcBAP* transgenic individuals among a number (30) of regenerated tobacco plantlets, as did for PtxD [24]. In addition, tobacco transformation, leaf explant regeneration test, Phi-resistance analysis of *EcBAP* transgenic tobaccos (on petri-dishes) and weed control simulation test (in greenhouse), were conducted with at least two replications.

## Supporting information

S1 Fig The diagram of plant transformation vector pBI(EcBAP). The *EcBAP* gene was subcloned in plant binary vector pBI121 between *CaMV 35S* promoter (termed Pro(CaMV 35S)) and *nos* terminator (termed Ter(nos)) by *BamH* I/*Sac* I digestions. The kanamycin (Kan)-selective marker gene *NPTII* was controlled by *nos* promoter (termed Pro(nos)) and Ter(nos).
(TIF)

S2 Fig Pairwise alignment of the clone sequencing-deduced EcBAP and the *E*. *coli phoA*-encoded protein. The N-terminal SP (1–21 aa) was truncated in the deduced protein of the cloned *EcBAP* gene. The codons of additional residues (MA in red letter) were introduced for initiating translation.
(TIF)

S3 Fig Generation of EcBAP(Kan) transgenic tobacco plants by Kan selection. (A) The procedure of tobacco transformation of plant vector pET(EcBAP) via Agrobacterium infiltration and Kan selection. (B) Identification of Kan-resistant *EcBAP* transgenic tobacco plantlets by multiplexed PCR with three primer-pairs EcBAP-5Bm/EcBAP-3Sc, EcBAP-5Bm/NosDw-Rv, and 35sPro-Fw/EcBAP-3Sc. M: DNA marker; Arrow-heads indicate the target PCR bands. The original gel image of this figure (B) is available in S1 File.
(TIF)

S4 Fig Leaf explant regeneration test of EcBAP(Kan) transgenic tobacco under high Phi stress. Small leaf pieces (0.5 cm × 0.5 cm) of WT and EcBAP(Kan) transgenic tobacco were pairwise laid on (A) MS (-Pi) or (B) standard MS medium, containing Phi of high concentrations (4, 5, 10 mM). After 7 days, 1 month, and even 2 months, the differentiation/regeneration status of these leaf explants under Phi stress were photo-recorded and compared between WT and EcBAP(Kan) transgenic tobacco.
(TIF)

S5 Fig Identification of Phi-selected *EcBAP* transgenic tobacco plants by multiplexed PCR. Experiments were performed using three primer-pairs, i.e. (A) EcBAP-5Bm/EcBAP-3Sc, (B) EcBAP-5Bm/ NosDw-Rv, and (C) 35sPro-Fw/ EcBAP-3Sc. M: DNA marker; Arrow-heads indicate the target PCR bands. The original gel image of this figure is available in S1 File.
(TIF)

S6 Fig RT-PCR analysis of *EcBAP* expression in the tissues of root, stem, and leaf of EcBAP(Phi) transgenic tobacco plants. (A) The extracted total RNA; (B) RT-PCR of *18S rRNA* (the internal reference gene) with a correct product (552 bp) by primer-pair Nt18S-iFw/Nt18S-iRv; (C) RT-PCR of *EcBAP* with a correct product (408 bp) by primer-pair EcBAP-iFw/EcBAP-iRv. R: root; S: stem; L: leaf; Arrow-heads indicate the target PCR bands. The original gel images of this figure (A–C) are available in S1 File.
(TIF)

S7 Fig Phi-tolerance test of weeds by judging the seed germination and seedling growth under Phi stress. Seeds of two selected weed species, Bermuda grass (W1) and Tall fescue (W2), were laid on the filter papers wetted with 0.1 x MS (–P, +80 mg·L^-1^ Pi, or +120 mg·L^-1^ Phi), and cultivated in a plant growth chamber under normal conditions for 20 days, then photo-recorded for both horizontal and vertical views.
(TIF)

S8 Fig Multiplexed PCR verification of the dominantly grown tobacco seedlings under Phi irrigation in the weed control simulation test. The blue-circled bigger seedlings in Fig 6 were substantiated as transgenic tobaccos by genomic PCR with three primer-pairs EcBAP-5Bm/EcBAP-3Sc, EcBAP-5Bm/NosDw-Rv, and 35sPro-Fw/EcBAP-3Sc. Arrow-heads indicate the target PCR bands. The original gel image of this figure is available in S1 File.
(TIF)

S9 Fig The framework diagram of this study. (TIF)

S1 File The original gel images. (DOCX)

S2 File Malachite green-based spectrometric assay to determine the Phi-oxidizing activity of recombinant EcBAP. (XLSX)
