# Supplementary figures and images for: A novel dominant selection system for plant transgenics based on phosphite metabolism catalyzed by bacterial alkaline phosphatase
Source: PLoS One. 2021 Nov 4;16(11):e0259600. doi: 10.1371/journal.pone.0259600 (PMC8568168; doi:10.1371/journal.pone.0259600)

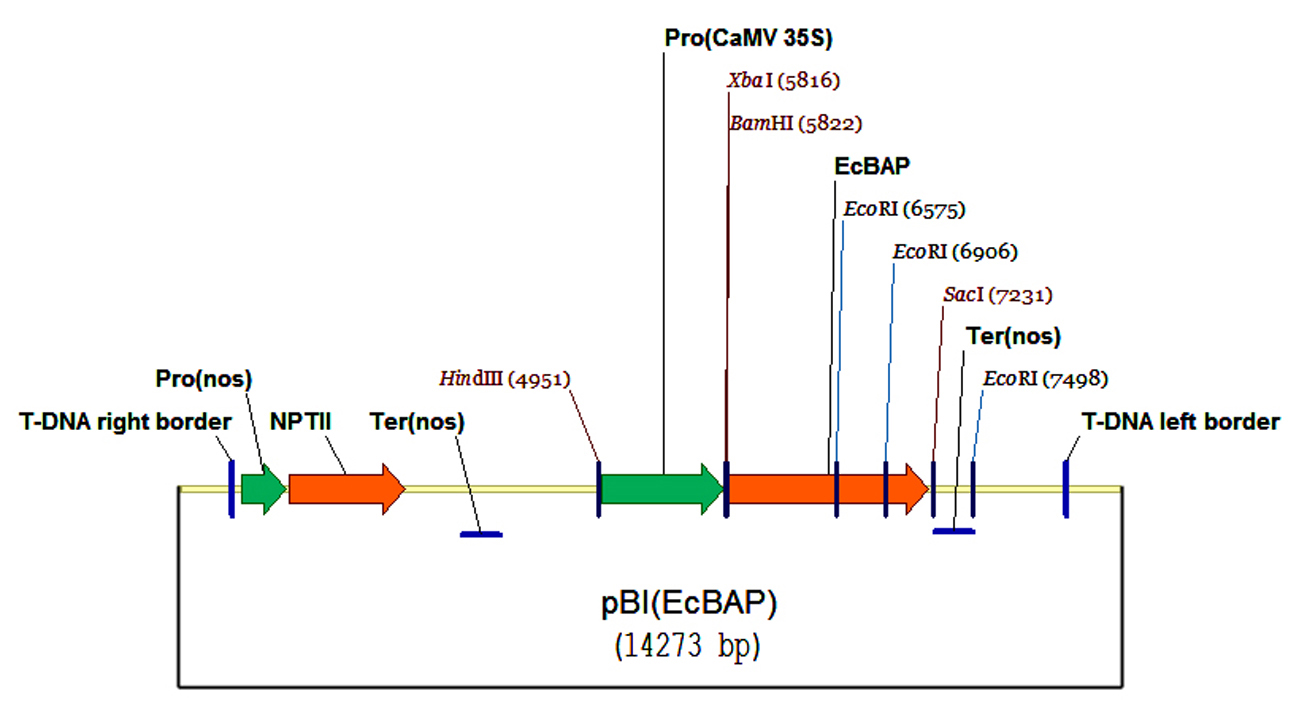

Supplement: S1 Fig — The EcBAP gene was subcloned in plant binary vector pBI121 between CaMV 35S promoter (termed Pro(CaMV 35S)) and nos terminator (termed Ter(nos)) by BamH I/Sac I digestions. The kanamycin (Kan)-selective marker gene NPTII was controlled by nos promoter (termed Pro(nos)) and Ter(nos). (TIF) [file pone.0259600.s001.tif]

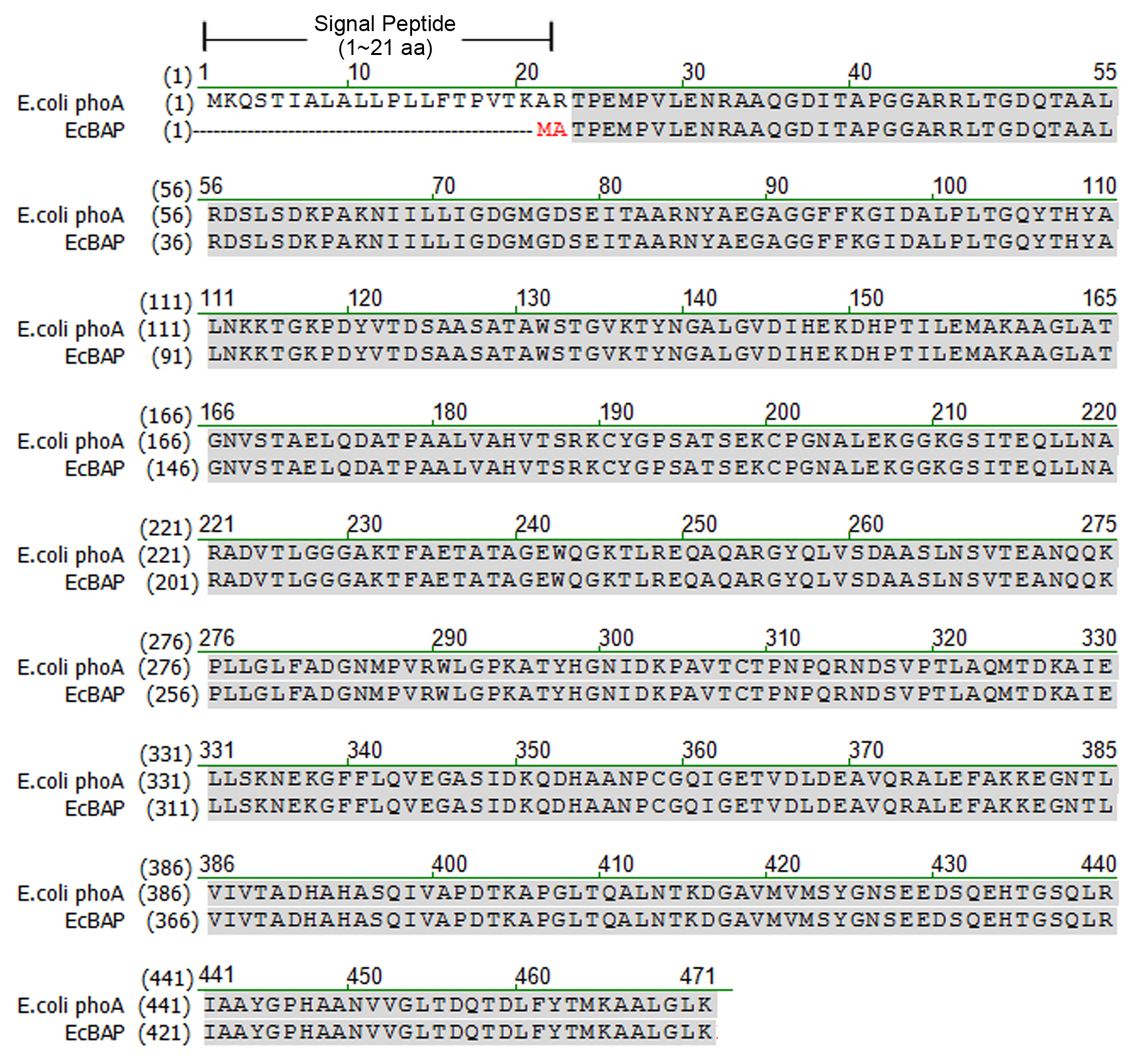

Supplement: S2 Fig — The N-terminal SP (1–21 aa) was truncated in the deduced protein of the cloned EcBAP gene. The codons of additional residues (MA in red letter) were introduced for initiating translation. (TIF) [file pone.0259600.s002.tif]

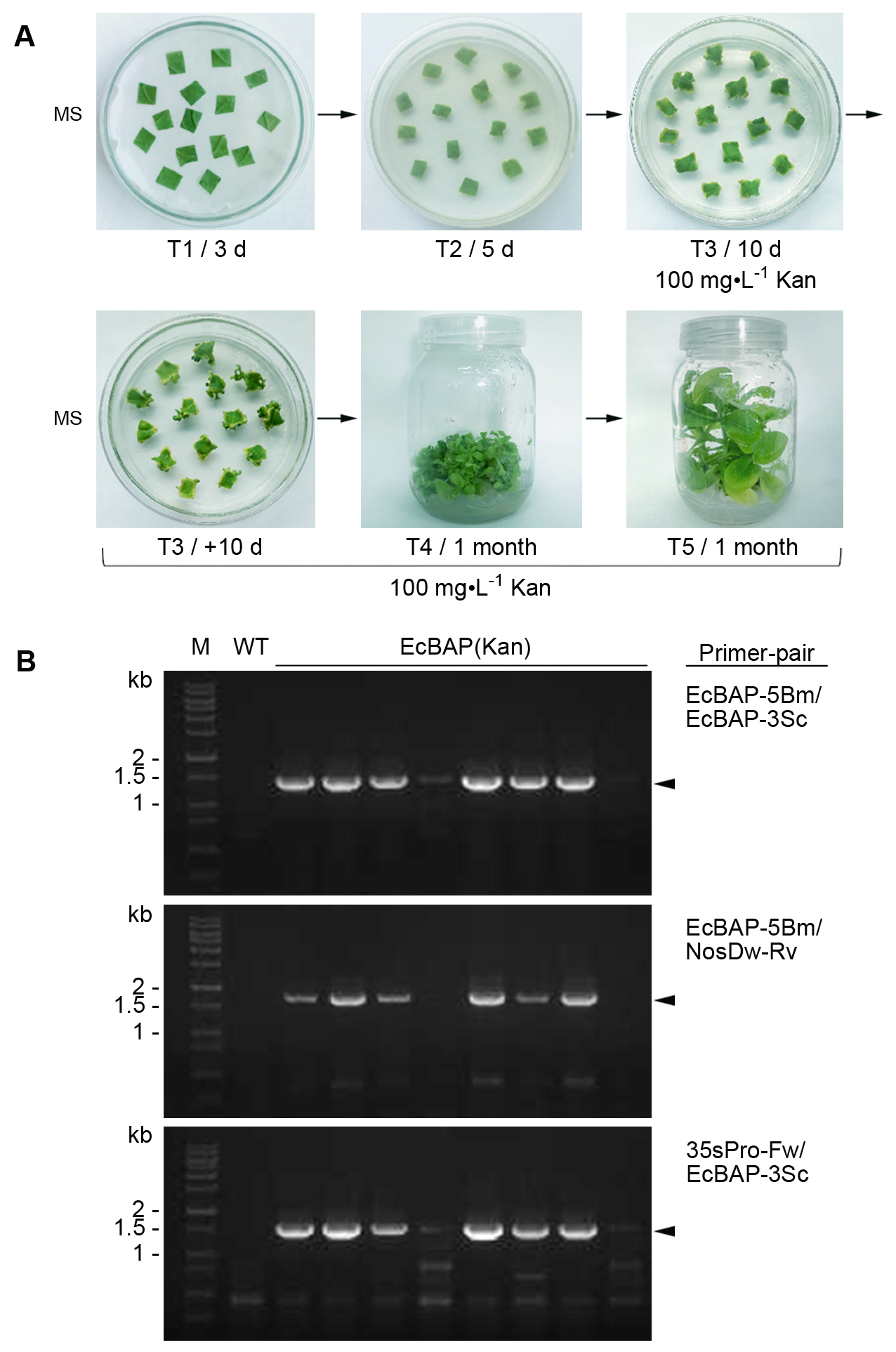

Supplement: S3 Fig — (A) The procedure of tobacco transformation of plant vector pET(EcBAP) via Agrobacterium infiltration and Kan selection. (B) Identification of Kan-resistant EcBAP transgenic tobacco plantlets by multiplexed PCR with three primer-pairs EcBAP-5Bm/EcBAP-3Sc, EcBAP-5Bm/NosDw-Rv, and 35sPro-Fw/EcBAP-3Sc. M: DNA marker; Arrow-heads indicate the target PCR bands. The original gel image of this figure (B) is available in S1 File. (TIF) [file pone.0259600.s003.tif]

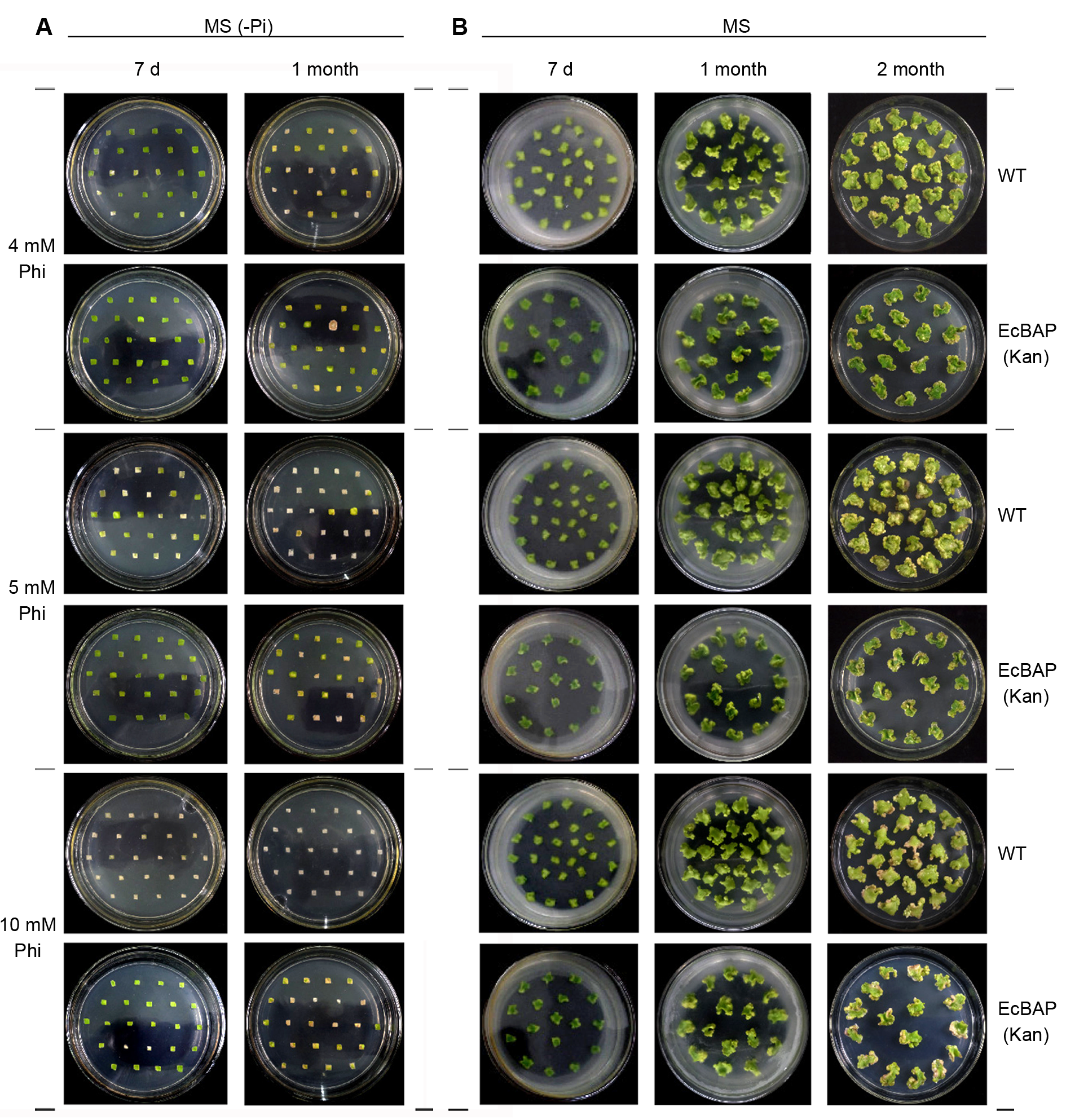

Supplement: S4 Fig — Small leaf pieces (0.5 cm × 0.5 cm) of WT and EcBAP(Kan) transgenic tobacco were pairwise laid on (A) MS (-Pi) or (B) standard MS medium, containing Phi of high concentrations (4, 5, 10 mM). After 7 days, 1 month, and even 2 months, the differentiation/regeneration status of these leaf explants under Phi stress were photo-recorded and compared between WT and EcBAP(Kan) transgenic tobacco. (TIF) [file pone.0259600.s004.tif]

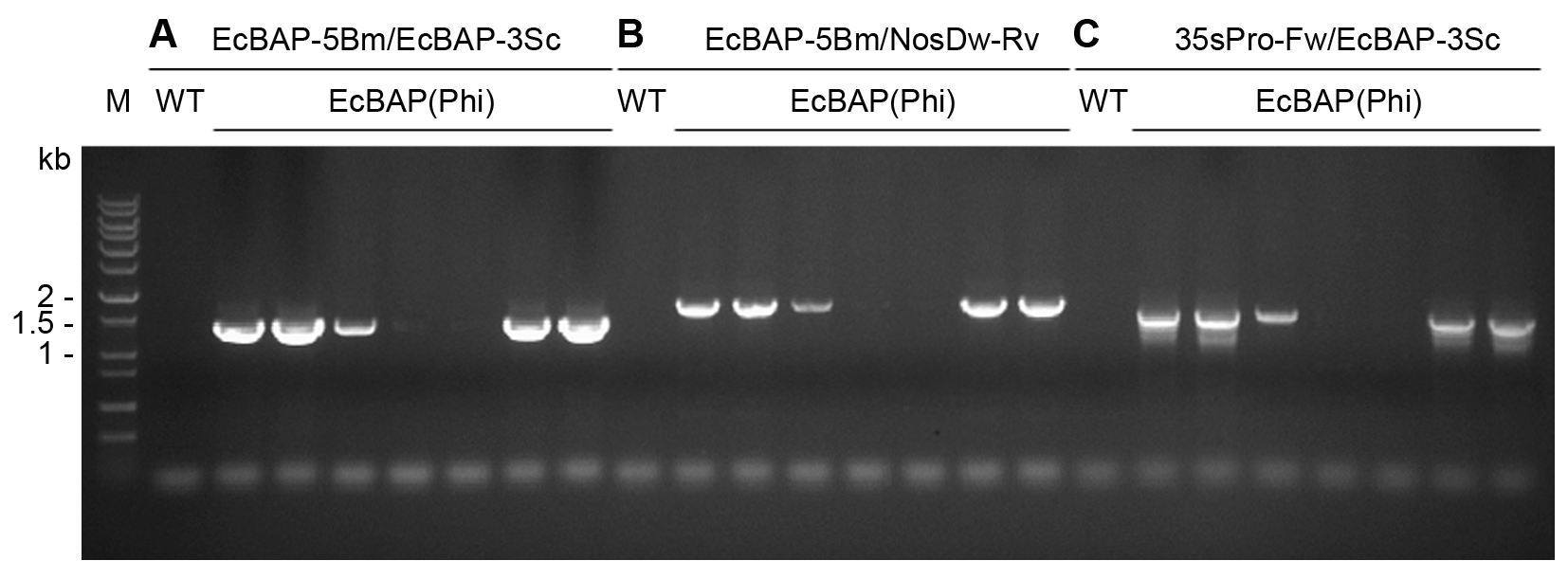

Supplement: S5 Fig — Experiments were performed using three primer-pairs, i.e. (A) EcBAP-5Bm/EcBAP-3Sc, (B) EcBAP-5Bm/ NosDw-Rv, and (C) 35sPro-Fw/ EcBAP-3Sc. M: DNA marker; Arrow-heads indicate the target PCR bands. The original gel image of this figure is available in S1 File. (TIF) [file pone.0259600.s005.tif]

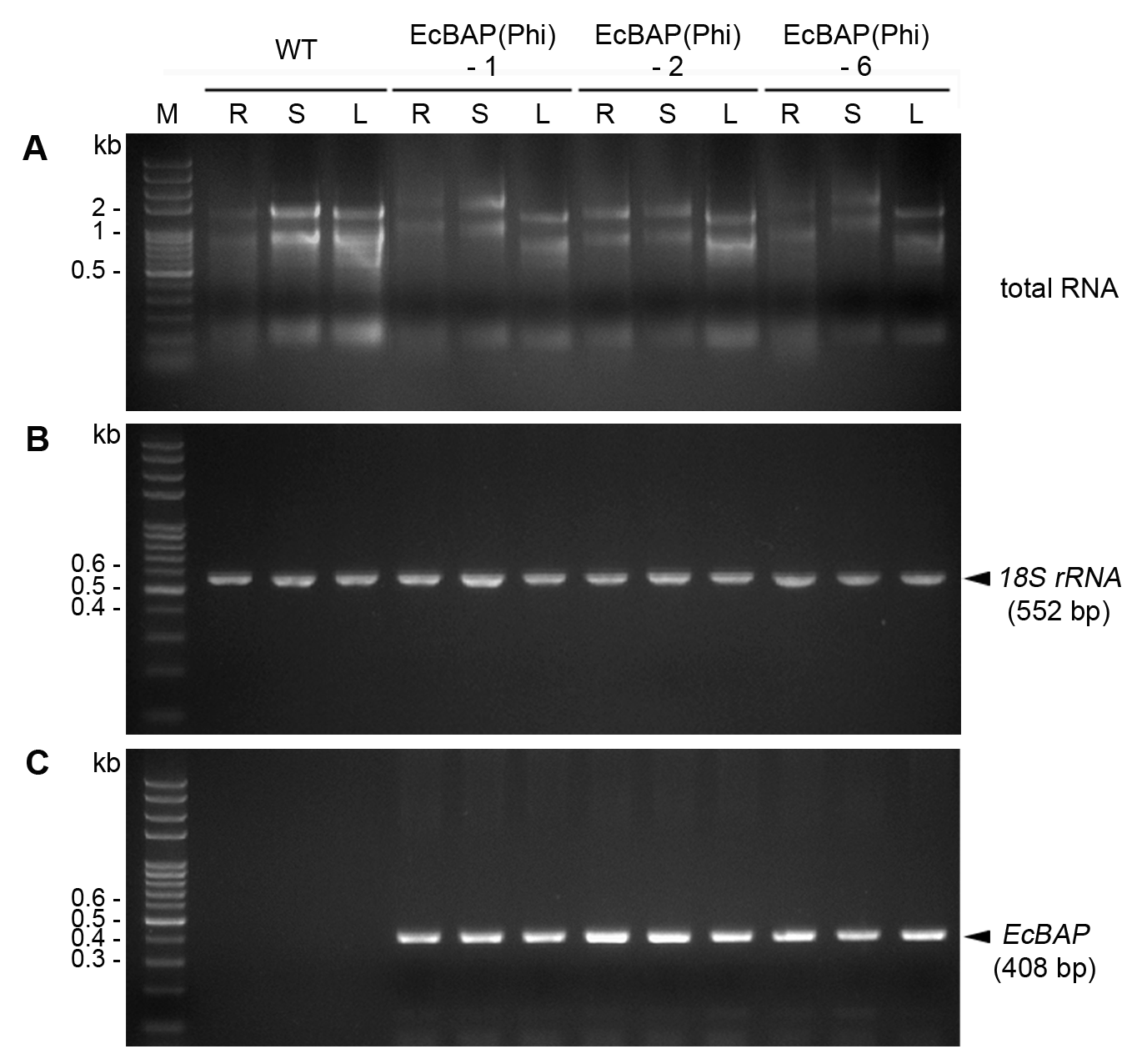

Supplement: S6 Fig — (A) The extracted total RNA; (B) RT-PCR of 18S rRNA (the internal reference gene) with a correct product (552 bp) by primer-pair Nt18S-iFw/Nt18S-iRv; (C) RT-PCR of EcBAP with a correct product (408 bp) by primer-pair EcBAP-iFw/EcBAP-iRv. R: root; S: stem; L: leaf; Arrow-heads indicate the target PCR bands. The original gel images of this figure (A–C) are available in S1 File. (TIF) [file pone.0259600.s006.tif]

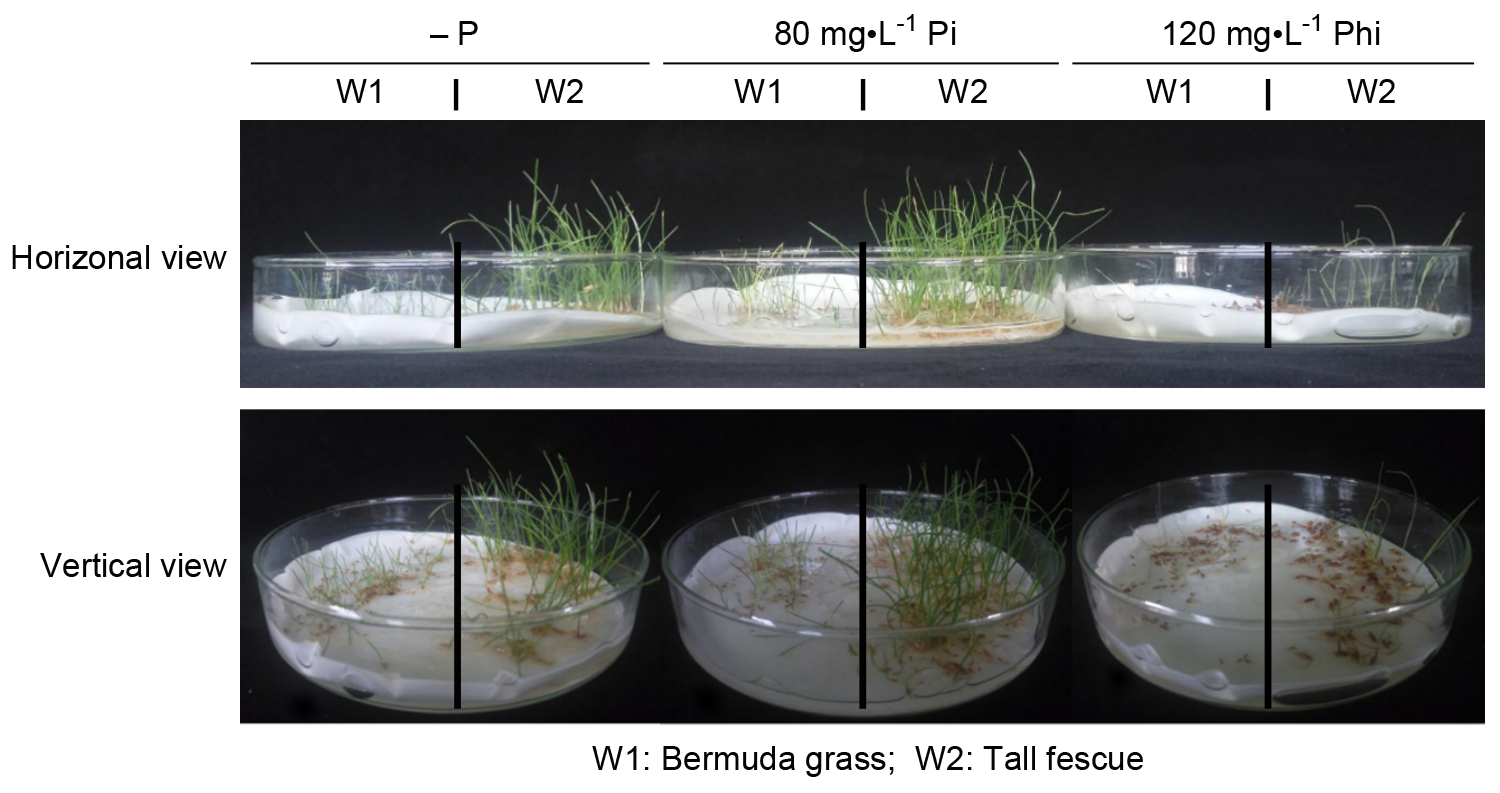

Supplement: S7 Fig — Seeds of two selected weed species, Bermuda grass (W1) and Tall fescue (W2), were laid on the filter papers wetted with 0.1 x MS (–P, +80 mg·L-1 Pi, or +120 mg·L-1 Phi), and cultivated in a plant growth chamber under normal conditions for 20 days, then photo-recorded for both horizontal and vertical views. (TIF) [file pone.0259600.s007.tif]

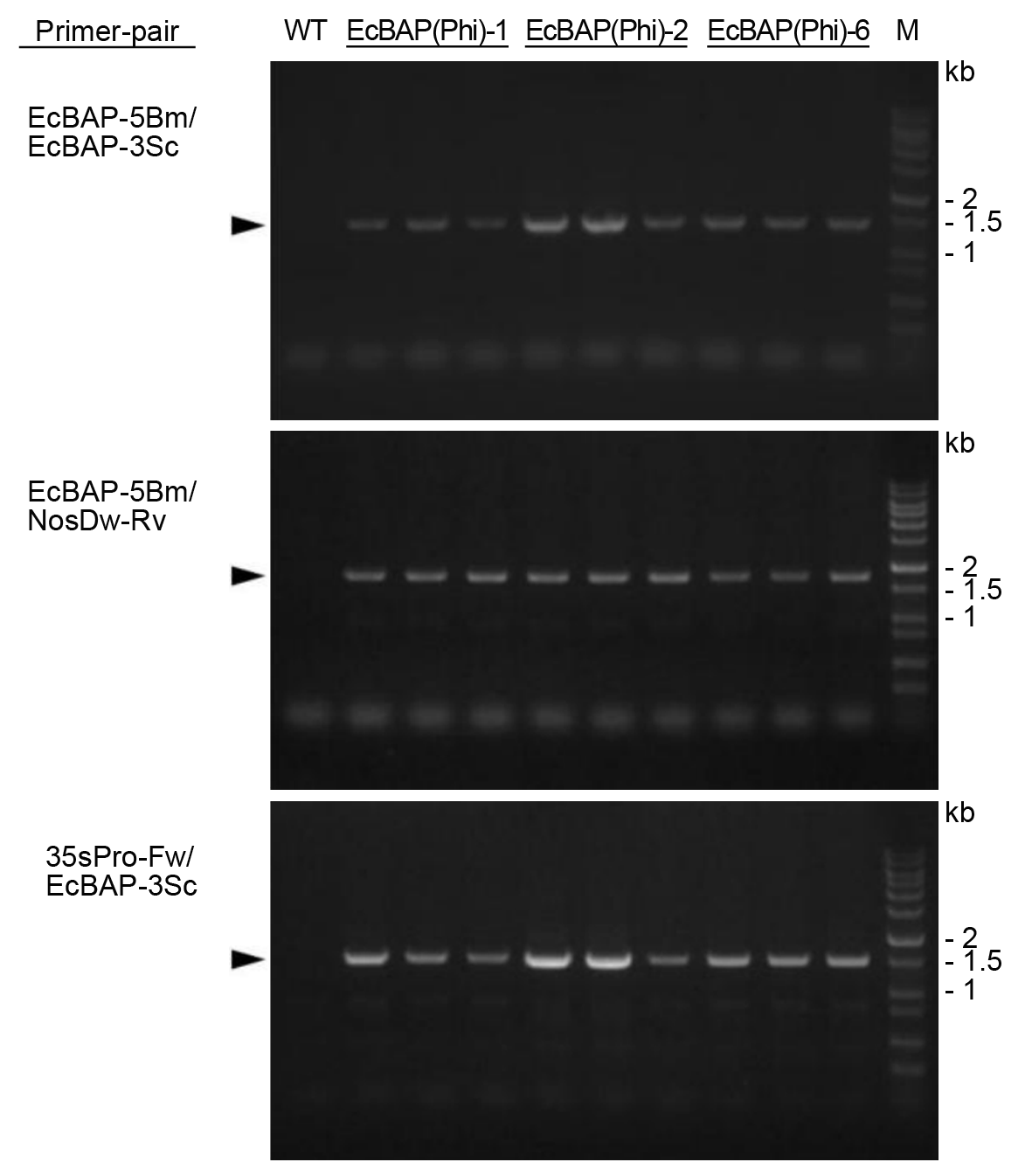

Supplement: S8 Fig — The blue-circled bigger seedlings in Fig 6 were substantiated as transgenic tobaccos by genomic PCR with three primer-pairs EcBAP-5Bm/EcBAP-3Sc, EcBAP-5Bm/NosDw-Rv, and 35sPro-Fw/EcBAP-3Sc. Arrow-heads indicate the target PCR bands. The original gel image of this figure is available in S1 File. (TIF) [file pone.0259600.s008.tif]

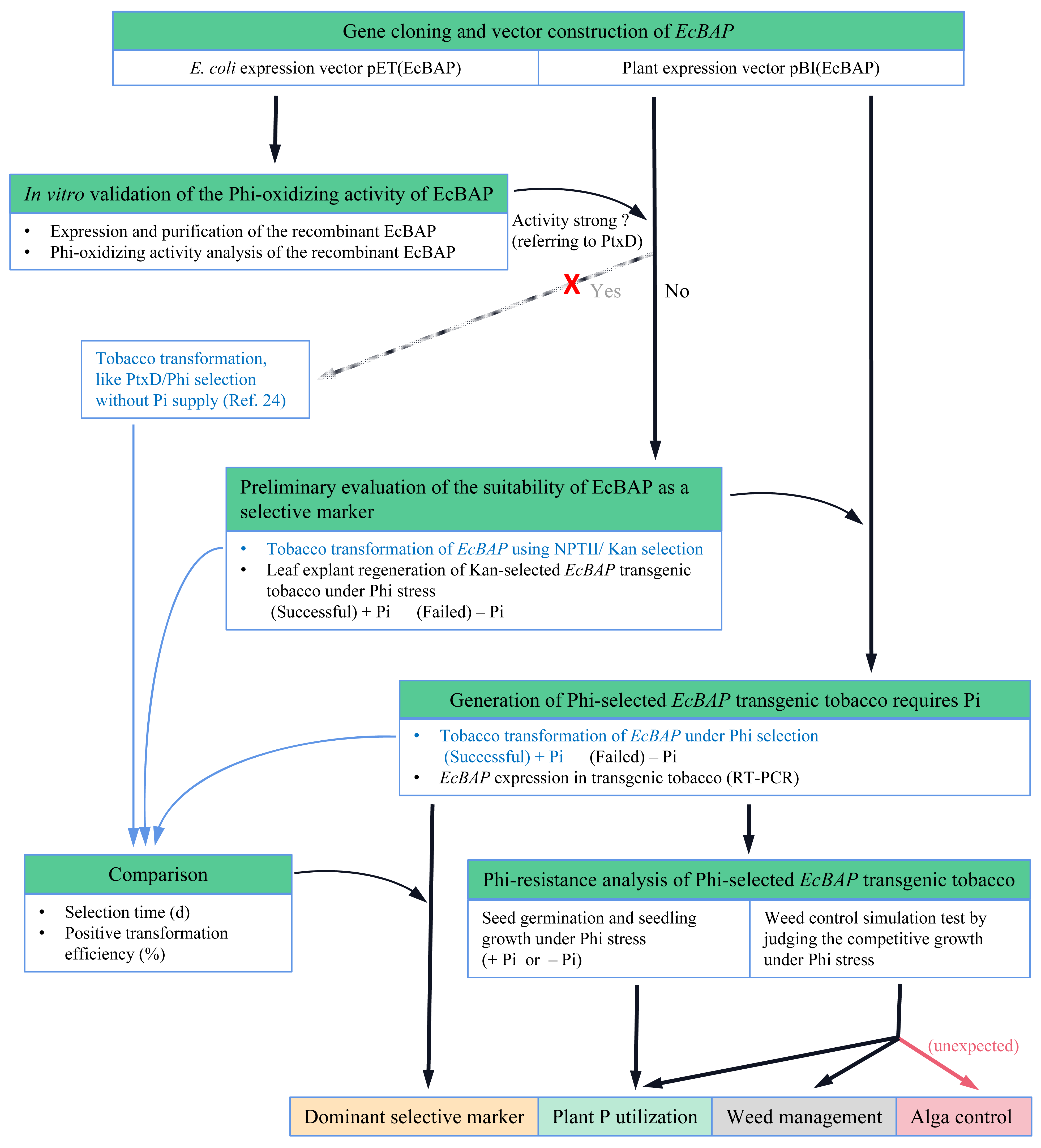

Supplement: S9 Fig — (TIF) [file pone.0259600.s009.tif]
