# Supplementary material for: A novel dominant selection system for plant transgenics based on phosphite metabolism catalyzed by bacterial alkaline phosphatase
Source: PLoS One. 2021 Nov 4;16(11):e0259600. doi: 10.1371/journal.pone.0259600 (PMC8568168; doi:10.1371/journal.pone.0259600)
Supplement: S1 File — (DOCX) [file pone.0259600.s010.docx]

**The original gel images of Fig 1.**

37 ºC/4 h

M UI T P S

**A** (boxed)

**
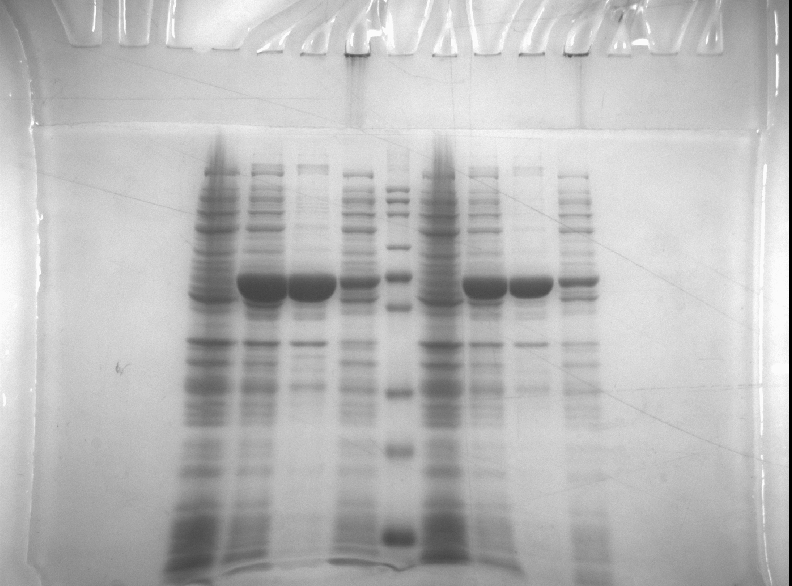
**

25 ºC/overnight

M UI T P S

**B** (boxed)

**
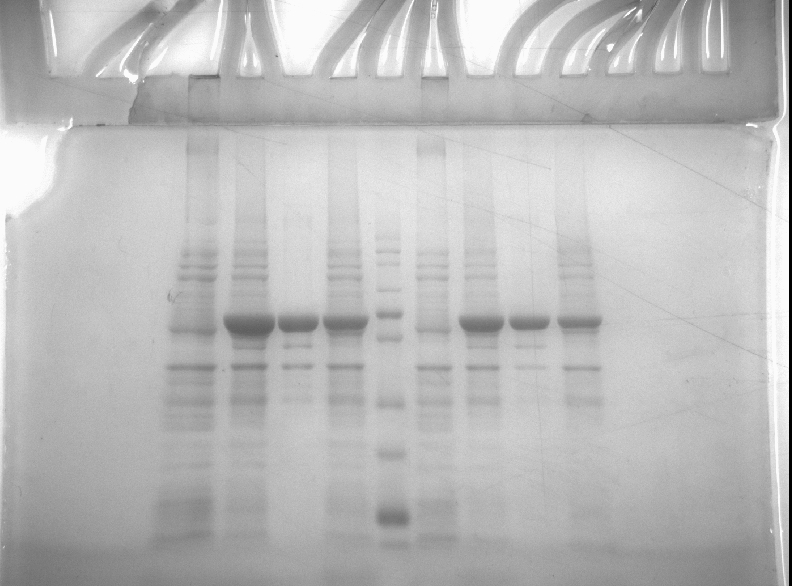
**

**The original gel images of Fig 2.**

EcBAP

M 1 2 3 (μg)

**
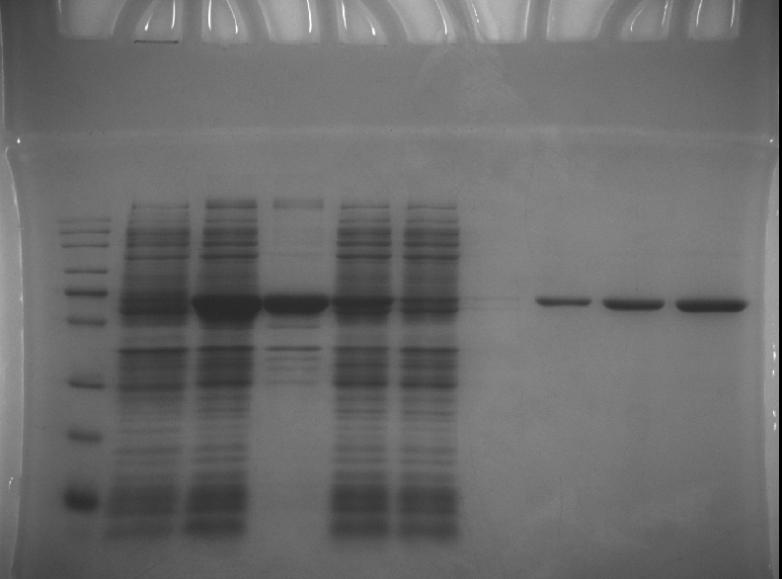
**

SDS-PAGE

(Coomassie blue)

**A** (boxed)

**
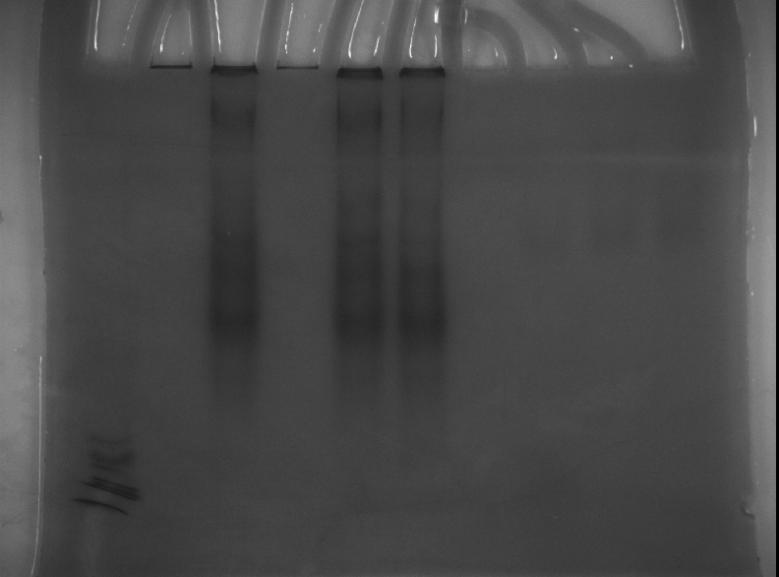
**

Native-PAGE

(Coomassie blue)

**B** (boxed)

**
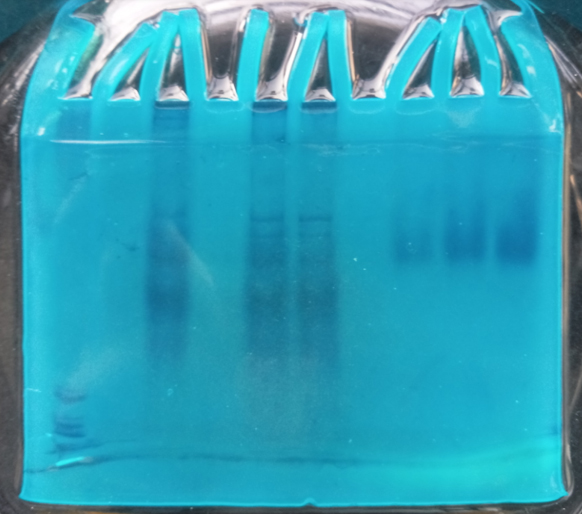
**

Native-PAGE

(Phi + Methyl green)

**C** (boxed)

**The original gel image of S3B Fig.**

M WT EcBAP(Kan) Primer-pair

**
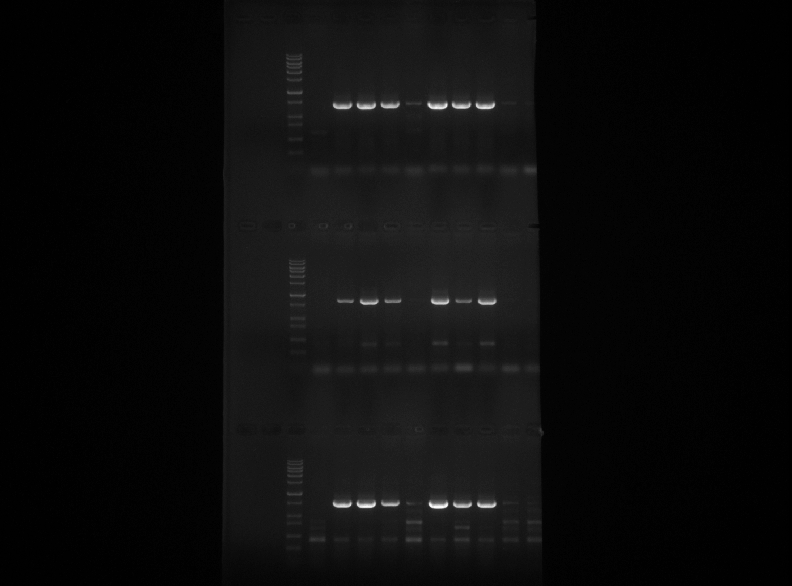
**

35sPro-Fw/

EcBAP-3Sc

EcBAP-5Bm/

EcBAP-3Sc

EcBAP-5Bm/

NosDw-Rv

**The original gel image of S5 Fig.**

**A** **B** **C**

EcBAP-5Bm/EcBAP-3Sc EcBAP-5Bm/NosDw-Rv 35sPro-Fw/EcBAP-3Sc

M WT EcBAP(Phi) WT EcBAP(Phi) WT EcBAP(Phi)

**
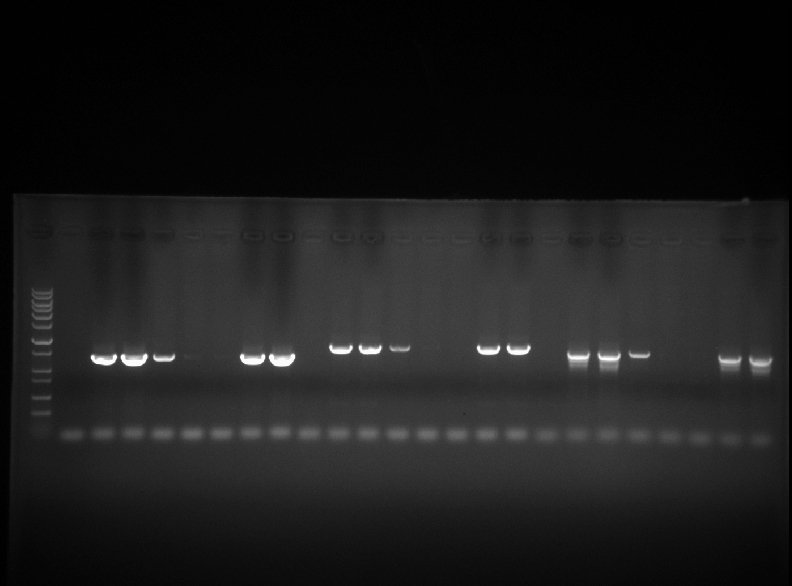
**

**The original gel images of S6 Fig.**

**
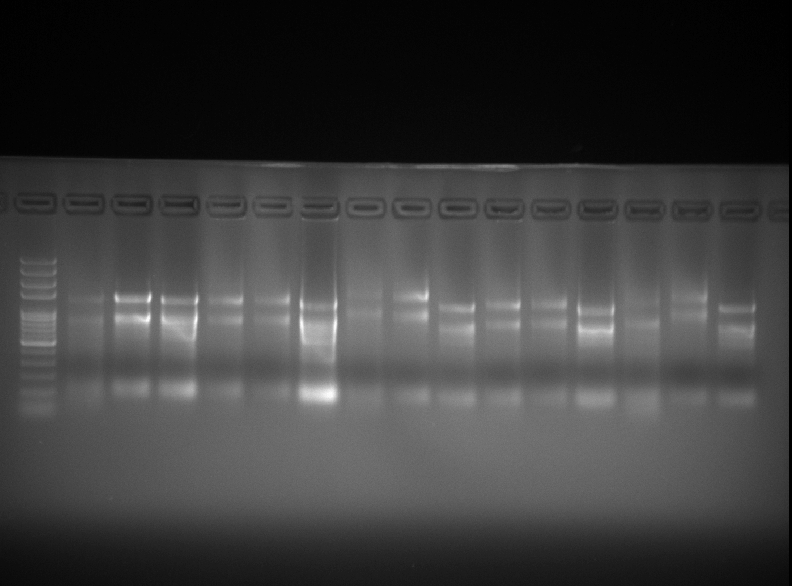
**

**A** (boxed)

total RNA

WT EcBAP(Phi)-1 EcBAP(Phi)-2 EcBAP(Phi)-6

M R S L R S L R S L R S L

M R S L R S L R S L R S L

WT EcBAP(Phi)-1 EcBAP(Phi)-2 EcBAP(Phi)-6

**
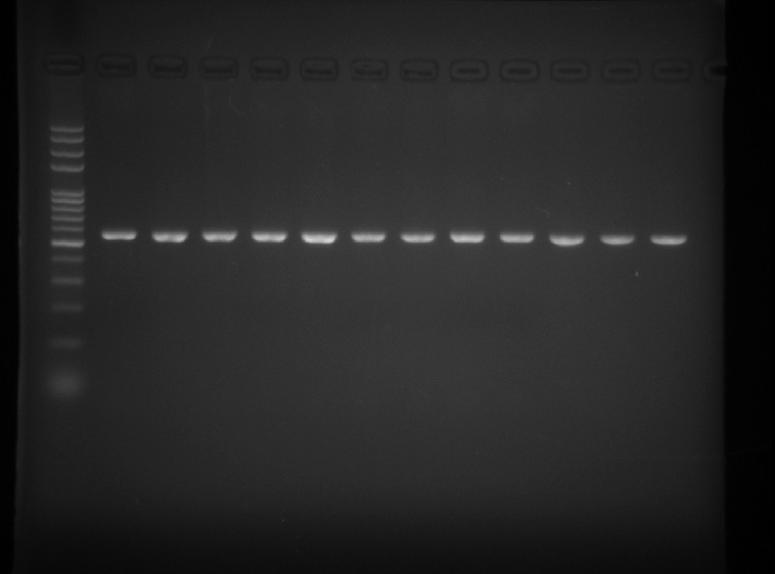
**

WT EcBAP(Phi)-1 EcBAP(Phi)-2 EcBAP(Phi)-6

**B**

*18S rRNA*

(552 bp)

M R S L R S L R S L R S L

**
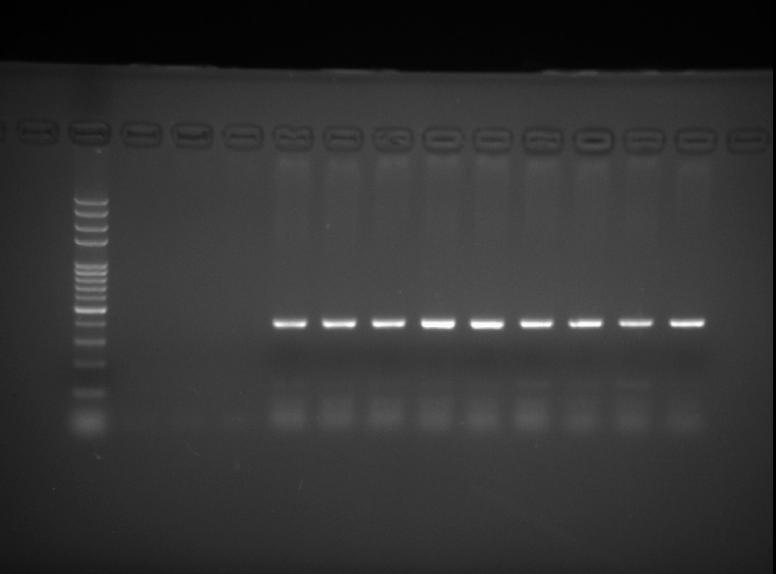
**

**C**

*EcBAP*

(408 bp)

**The original gel image of S8 Fig.**

Primer-pair WT EcBAP(Phi)-1 EcBAP(Phi)-2 EcBAP(Phi)-6 M


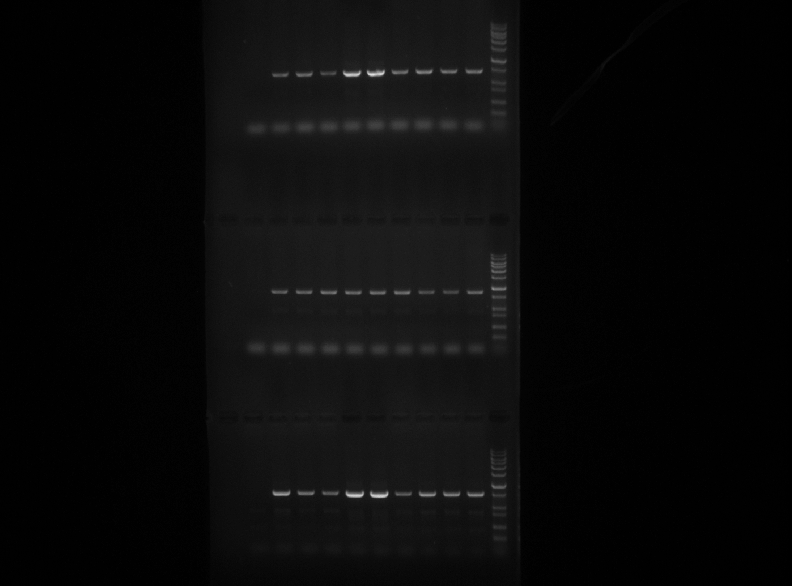


EcBAP-5Bm/

NosDw-Rv

35sPro-Fw/

EcBAP-3Sc

EcBAP-5Bm/

EcBAP-3Sc
